# Nucleic acid recognition and antiviral activity of 1,4-substituted terphenyl compounds mimicking all faces of the HIV-1 Rev protein positively-charged α-helix

**DOI:** 10.1038/s41598-020-64120-2

**Published:** 2020-04-28

**Authors:** Cristina Medina-Trillo, Daniel M. Sedgwick, Lidia Herrera, Manuela Beltrán, Ángela Moreno, Pablo Barrio, Luis. M. Bedoya, José Alcamí, Santos Fustero, José Gallego

**Affiliations:** 1 Facultad de Medicina y Odontología, Universidad Católica de Valencia San Vicente Mártir, C/Quevedo 2, 46001 Valencia, Spain; 2 Departamento de Química Orgánica, Universidad de Valencia, Avda. V. Andrés Estellés s/n, 46100 Burjassot, Valencia Spain; 3 Unidad de Inmunopatología del SIDA, Centro Nacional de Microbiología, Instituto de Salud Carlos III, Carretera Majadahonda-Pozuelo km 2, 28220 Majadahonda, Madrid Spain; 4 Departamento de Farmacología, Famacognosia y Botánica, Facultad de Farmacia, Universidad Complutense de Madrid, Pz. Ramón y Cajal s/n, 28040 Madrid, Spain; 5 Infectious Diseases Unit, IBIDAPS, Hospital Clínic, Universidad de Barcelona, C/Roselló, 149 08036 Barcelona, Spain; 6 Centro de Investigación Príncipe Felipe, Avda. Autopista del Saler 16, 46012 Valencia, Spain

**Keywords:** HIV infections, Small molecules, Target identification, Mechanism of action, RNA, Drug discovery, Antivirals, Retrovirus

## Abstract

Small synthetic molecules mimicking the three-dimensional structure of α-helices may find applications as inhibitors of therapeutically relevant protein-protein and protein-nucleic acid interactions. However, the design and use of multi-facial helix mimetics remains in its infancy. Here we describe the synthesis and application of novel bilaterally substituted *p*-terphenyl compounds containing positively-charged aminoalkyl groups in relative 1,4 positions across the aromatic scaffold. These compounds were specifically designed to mimic all faces of the arginine-rich α-helix of the HIV-1 protein Rev, which forms deeply embedded RNA complexes and plays key roles in the virus replication cycle. Two of these molecules recognized the Rev site in the viral RNA and inhibited the formation of the RRE-Rev ribonucleoprotein complex, a currently unexploited target in HIV chemotherapy. Cellular assays revealed that the most active compounds blocked HIV-1 replication with little toxicity, and likely exerted this effect through a multi-target mechanism involving inhibition of viral LTR promoter-dependent transcription and Rev function. Further development of this scaffold may open new avenues for targeting nucleic acids and may complement current HIV therapies, none of which involve inhibitors interfering with the gene regulation processes of the virus.

## Introduction

**Figure 1:**
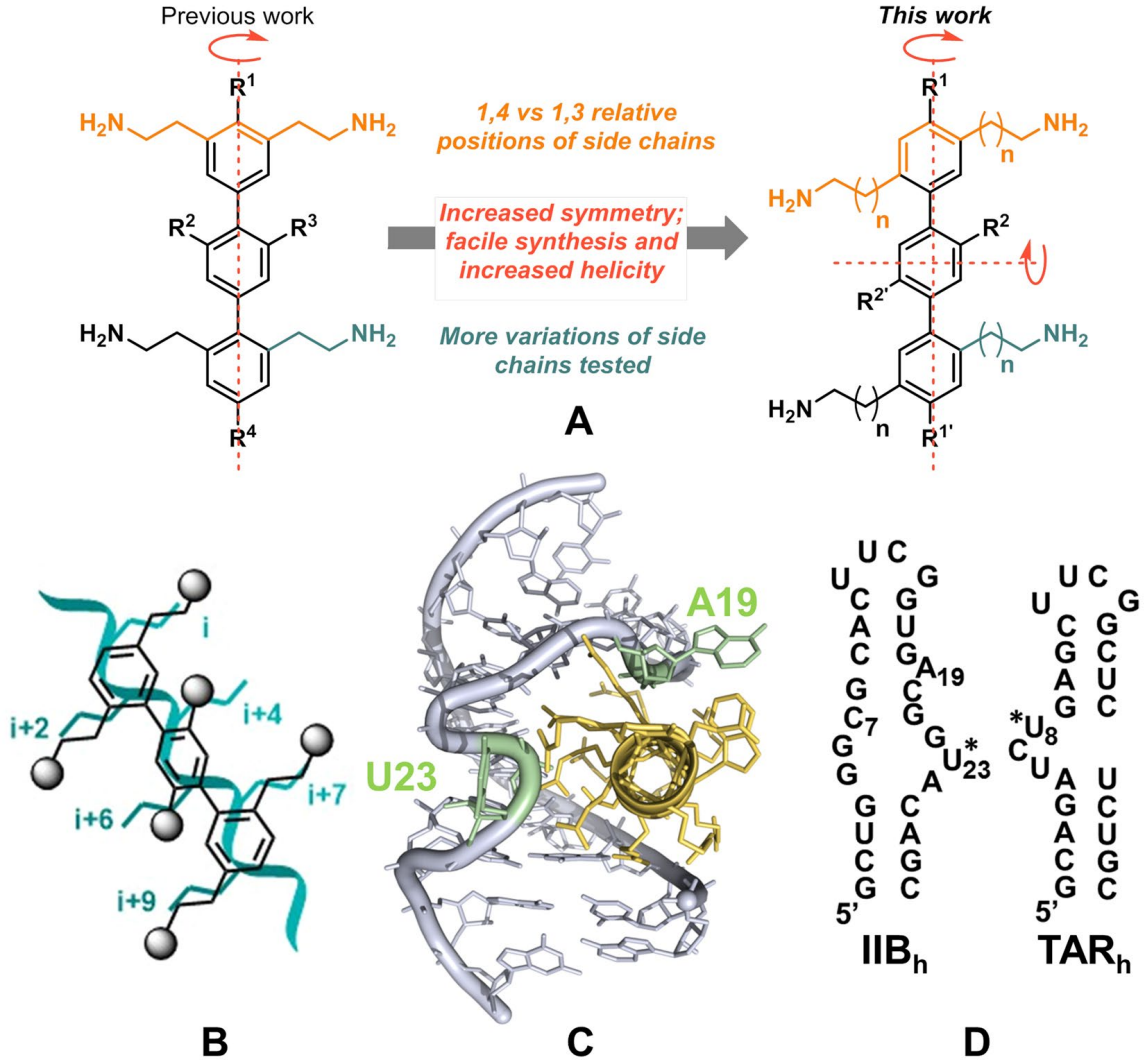
(**A**) Comparison of bilateral terphenyls with side chains in relative 1,3 (left) and 1,4 (right) positions. In both cases, the aminoalkyl side chains imitate the Arg residues of Rev. (**B**) Schematic overlay of a 1,4-bilaterally substituted terphenyl and a protein α-helix showing the mimicked residues (cyan). (**C**) Three-dimensional view of the complex formed between subdomain IIB of the RRE (grey) and the Rev_34–50_ helix of the HIV-1 protein Rev (yellow)^12^. The extrahelical loop residues A19 and U23 are coloured light green. The image was generated with MOE 2019.0102 (www.chemcomp.com). (**D**) Secondary structure of RNA hairpins IIB_h_, containing the high-affinity Rev_34–50_ binding site, and TAR_h_, used as a specificity control. For fluorescence intensity assays a fluorescein probe was linked to the extra-helical loop residues U23 of IIB_h_ and U8 of TAR_h_ (indicated with asterisks).

Protein α-helices are often involved in interactions with DNA, RNA or other proteins^1^. These complexes regulate many important biological processes, but are widely considered difficult targets for drug development. In this context, there has been a strong interest in developing synthetic small molecules that mimic the topology of α-helices, as this would facilitate the drug discovery process while potentially overcoming the pharmacokinetic limitations often encountered when using peptides as drugs^2–4^. Hamilton *et al*. pioneered this field by reporting that tris-substituted 3,2′,2″-terphenyl molecules reproduced the angular orientation of side chains i, i + 4 and i + 7 of an α-helix, and were capable of blocking protein-protein interactions^5,6^. However, Hamilton’s design was restricted to terphenyls substituted on one side of the molecule, mimicking just one face of an α-helix and limiting the possible therapeutic applications to superficial protein-protein interactions. We recently reported that terphenyl molecules with bilateral 3,5, 2′,6′,2″,6″ substitutions adopted a staggered conformation that matched the projection of side chains i, i + 1. i + 4, i + 5, i + 7 and i + 8, thereby mimicking all three faces of an α-helix and opening up the possibility of mimicking interactions in which the helix is deeply embedded in its receptor (Fig. 1)^7^. One such interaction is formed between the RNA-binding α-helix of the HIV-1 protein Rev and the virus RNA. The Rev protein adopts a helix-turn-helix conformation^8–10^, and the Nt-segment of the second helix contains a positively-charged arginine-rich motif, T_34_RQARRNRRRRWRERQR_50_ (hereafter identified as Rev_34–50_) that drives RNA-binding and also functions as a nuclear localization signal (NLS). The Rev_34–50_ helix forms a high-affinity interaction with an internal loop located within subdomain IIB of the Rev Recognition Element (RRE) of the viral RNA (Fig. 1C)^11,12^. This interaction is essential for virus viability, as it triggers the cooperative incorporation of additional Rev molecules into the complex through interactions between Rev_34–50_ helices and further sites on the RRE as well as protein-protein contacts^13^, allowing nuclear export of unspliced or singly-spliced viral RNA molecules in the late phase of the virus cycle^14^. Nevertheless, despite its importance in the viral replication cycle, the RRE-Rev ribonucleoprotein complex remains an unexploited target for HIV-1 chemotherapy. While a number of small-molecule compounds with substantial anti-HIV activity have been reported to block Rev function, most of them do not directly inhibit the formation of the RRE-Rev complex, or were found to bind to Rev partners in the host cell such as Crm1 or the cap-binding complex^15–19^.

In this work, we explore the use of bilateral terphenyl molecules containing substitutions in relative 1,4 positions across the *p*-terphenyl scaffold. Relative to the previously reported 1,3-terphenyl compounds^7^, these molecules match a different set of α-helix side chains and offer the major advantage of a simplified synthetic route due to increased symmetry (Fig. 1A). The most active compounds successfully inhibited the formation of both the RRE IIB- Rev_34–50_ and full-length RRE-Rev complexes, and blocked HIV-1 replication with little cellular toxicity. Furthermore, a detailed analysis of RNA recognition properties and cellular effects revealed that these molecules likely act through a multi-target mechanism involving inhibition of RNA transcription and Rev function.

## Results

### Synthesis of 1,4-terphenyl compounds

In terms of synthesis, the major advantage that this series of terphenyl compounds presented over the previously described 1,3 series was the complete symmetry of the side chains. Consequently, the synthesis was drastically simplified in two ways: (i) the two terminal phenyl rings were structurally equivalent, therefore the synthesis of an entire separate phenyl ring was saved; and (ii) just one Suzuki-Miyaura cross-coupling step was required to construct the desired terphenyl scaffold, rather than two subsequent coupling steps seen in the previous 1,3 series^7^. Hence, the synthesis of the final compounds consisted in the prior preparation of just two key synthons. The terminal synthons were aryl bromides bearing nitrile groups on the two side chains as masked amines, ready to be revealed in later steps, whereas the central synthon presented two alkyl side chains and two boronic esters. A simple double palladium-catalyzed Suzuki-Miyaura cross-coupling between two terminal synthons to every one central synthon constructed the desired terphenyl scaffold, and a subsequent borane-mediated reduction of the nitrile groups resulted in the amines required to mimic the arginine residues in Rev_34–50_ (Fig. S1).

**Figure 2:**
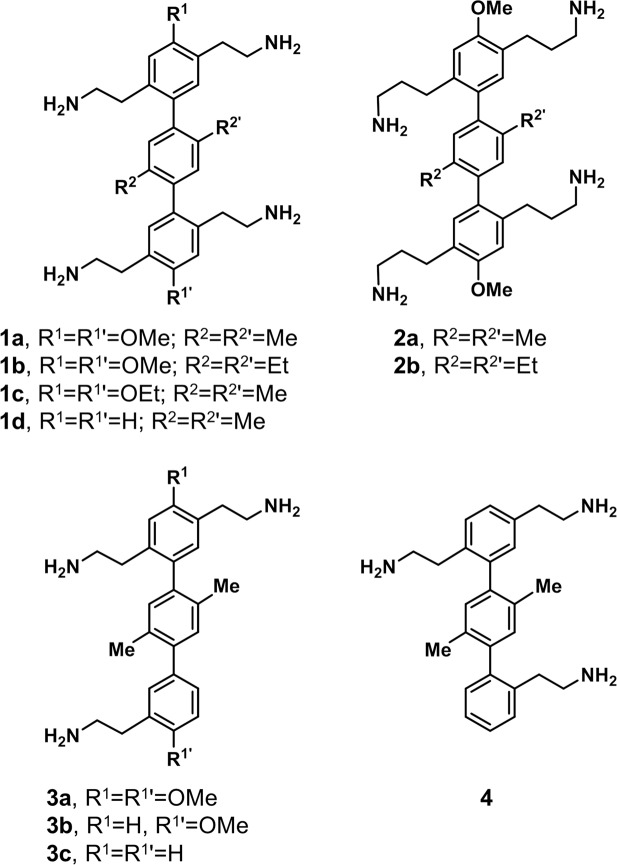
Chemical structure of 1,4-substituted *p*-terphenyl compounds analysed in this study.

Following this methodology, a library of terphenyl compounds bearing bilateral 1,4-side groups was generated. Within this library, we explored varying the length of the aminoalkyl side chains (terphenyl compounds **1** vs **2**), as well as different substitutions in the pole positions and different alkyl substitutions on the central phenyl ring (terphenyls **1a-d**). Furthermore, the effect of lowering the positive charge of the compounds was also investigated with terphenyls **3** and **4**, which contained just three aminoalkyl side chains in varying positions (Fig. 2).

### Inhibition of the RRE-Rev interaction

Molecular modelling calculations indicated that 1,4-terphenyl molecules could approximately match side chains i, i + 2, i + 4, i + 6, i + 7 and i + 9 of an α-helix (Fig. 1B). When applied to the Rev_34–50_ α-helix, the side chains of our terphenyl compounds were found to coincide with Rev residues reported to be essential for the interaction with subdomain IIB (Fig. S2).

**Figure 3:**
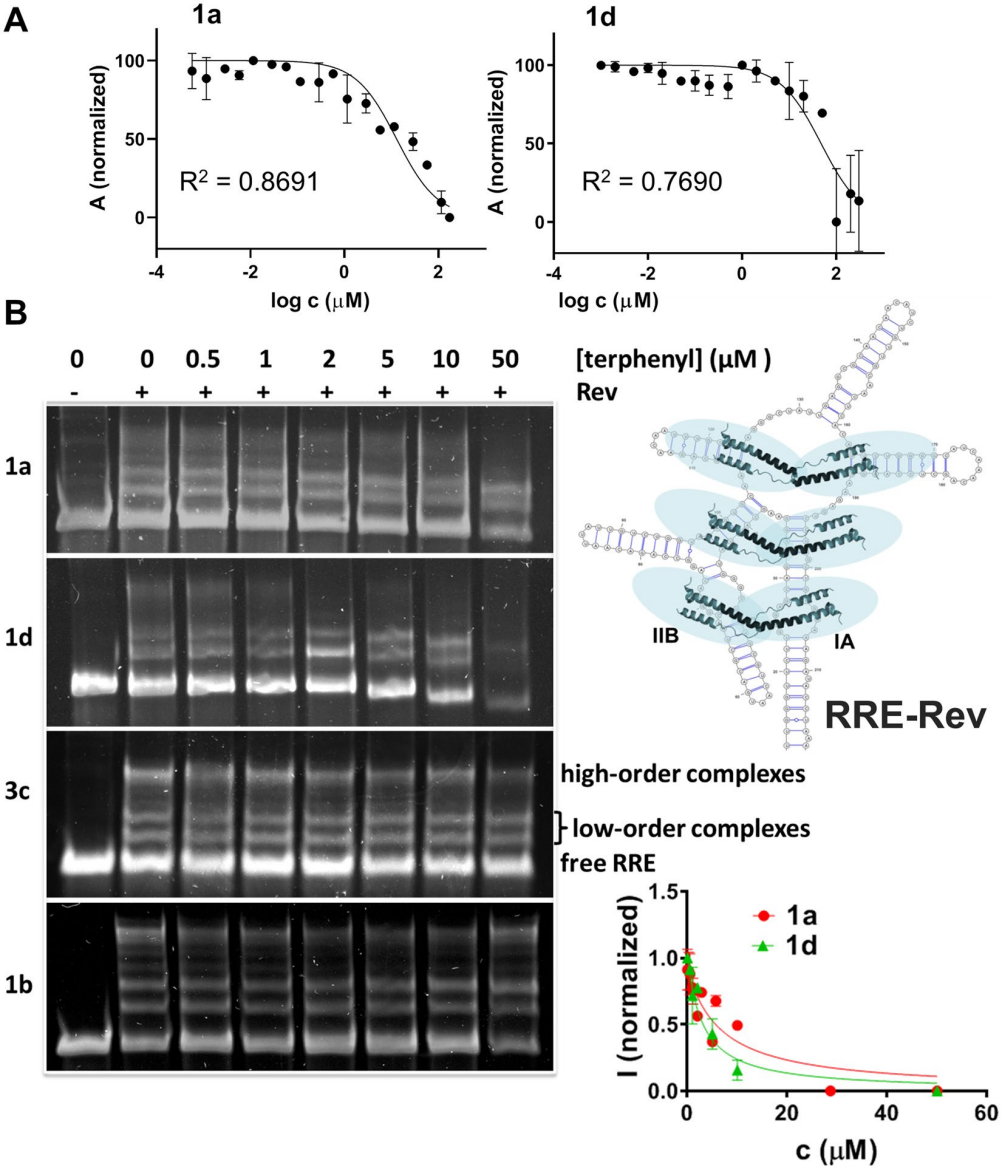
Inhibition of subdomain IIB-Rev_34–50_ and full-length RRE–Rev complex formation by 1,4-terphenyl compounds. (**A**) Curves of IIB_h_-Rev_34–50_ inhibition by terphenyls **1a** and **1d**, as measured by fluorescence anisotropy. (**B**) Inhibition of the RRE-Rev ribonucleoprotein complex by terphenyl compounds, analysed by EMSA experiments. The gel lanes contained 78 nM RRE, 1.32 μM Rev (marked with +) and the specified concentrations of compound **1a**, **1d**, **3c** and **1b**. The image shows on the right a schematic representation of the RRE-Rev ribonucleoprotein, with the location of the main Rev binding site in subdomain IIB indicated and the Rev monomers represented with ribbons and blue ovals, together with plots of high-order RRE-Rev band intensity as a function of terphenyl **1a** and **1d** concentration; the error bars represent the standard deviation of three independent experiments.

We first evaluated whether 1,4-terphenyl molecules were capable of inhibiting the high-affinity interaction between Rev_34–50_ and RRE subdomain IIB by using a displacement experiment based on fluorescence anisotropy^7,20^. Terphenyls **1a** and **1d** inhibited the IIB_h_-Rev_34–50_ contact with IC_50_ values of 14 and 47 μM respectively. The remaining terphenyl compounds had weaker or non-detectable activity at the assay concentrations (Figs. 3A and S3 and Table 1).

**Table 1:** 50% inhibitory concentrations of 1,4-terphenyl molecules for RRE subdomain IIB-Rev_34–50_ and high-order RRE-Rev complex formation.

Compound	IC_50_, RRE subdomain IIB-Rev_34–50_ (μM)^a^	IC_50_, high-order RRE-Rev (μM)^b^
**1a**	14 (9.0–20, 0.8691)	6.8 (3.8–12, 0.8337)
**1b**	>100	>50
**1c**	>100	n/d
**1d**	47 (33–68, 0.7690)	3.0 (2.0–4.7, 0.9242)
**2a**	>100	n/d
**2b**	>100	n/d
**3a**	>100	n/d
**3b**	>100	n/d
**3c**	>100	>50
**4**	>100	n/d

^a^RRE subdomain IIB–Rev_34–50_ IC_50_ values were obtained with fluorescence anisotropy experiments using 60 nM IIB_h_ and 10 nM frevp in the presence of 100 mM KCl, 20 mM NaCl and 2 mM MgCl_2_. ^b^Full-length RRE–Rev IC_50_ values were measured by EMSA with 78 nM RRE, 1.32 μM Rev, 300 mM KCl and 1 mM MgCl_2_, analysing the band corresponding to high-order complexes. The table shows best-fit IC_50_ values, with 95% confidence intervals and R^2^ coefficients shown in parentheses when applicable. n/d: not determined.

After the high-affinity interaction between RRE subdomain IIB and the Rev_34–50_ helix of the first Rev monomer is established, the RRE-Rev ribonucleoprotein is formed by the incorporation of additional Rev units binding to further sites in the RRE (Fig. 3B, right)^9,10,13^. Using an electrophoretic mobility shift assay (EMSA) involving full-length RRE and Rev, we also evaluated whether 1,4-terphenyl compounds were capable of interfering with the formation of the ribonucleoprotein. The results indicated that compounds **1a** and **1d** inhibited the RRE-Rev interaction (Fig. 3B). The effect was particularly prominent for high-order complexes (containing a greater number of Rev monomers), but the inhibition of low-order complexes by **1d** was also detected at concentrations consistent with the IC_50_ value measured in the IIB-Rev_34–50_ displacement experiment. Compounds **1b** and **3c** exerted a weaker effect on the RRE-Rev complex, in agreement with the results obtained in the displacement experiments monitored by fluorescence anisotropy (Fig. 3B and Table 1).

### RRE subdomain IIB RNA recognition

**Figure 4:**
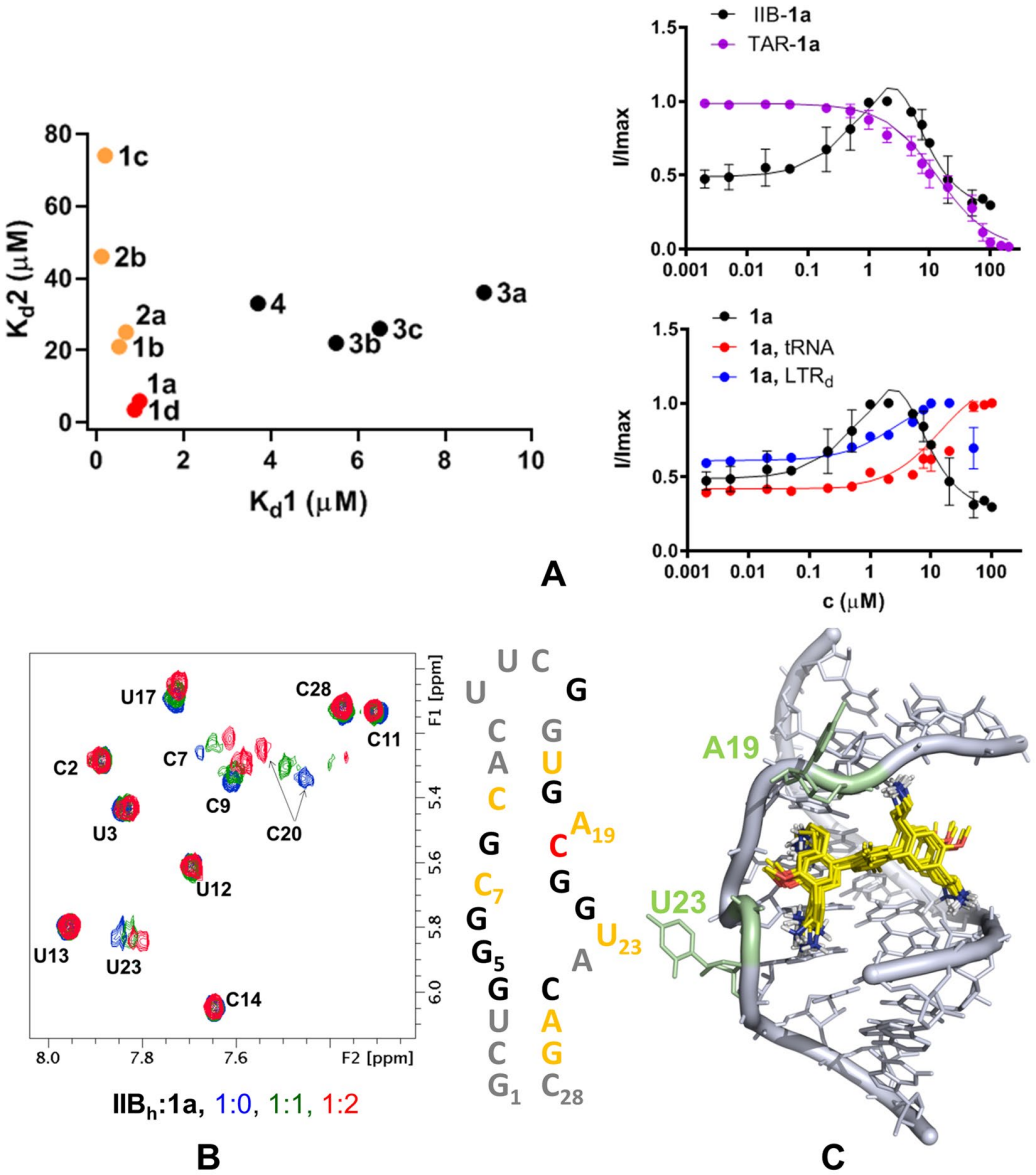
RRE subdomain IIB recognition by 1,4-terphenyl compounds. (**A**) Results of fluorescence intensity experiments. Left: comparison between first-site (K_d_1) and second-site (K_d_2) equilibrium dissociation constants for the interaction between IIB_h_ and 1,4-terphenyl molecules. Right: comparison of the IIB_h_ binding curve of terphenyl **1a** (black) with the TAR_h_ association curve (magenta), and with IIB_h_ binding curves obtained in the presence of a 10-fold molar excess of unlabelled competitor RNA (tRNA^Lys^; red) or unlabelled competitor double-helical DNA (LTR_d_; blue). Solution conditions: 10 mM sodium phosphate pH 6.6 and 0.1 mM EDTA. (**B**) Titration of IIB_h_ with terphenyl **1a** monitored by NMR spectroscopy. The H5-H6 region of the TOCSY spectrum of unbound IIB_h_ (blue) is superposed on the spectra of complexes with increasing RNA:**1a** molar ratios, color-coded as indicated in the graph. A map of the **1a** binding site in the IIB_h_ hairpin is shown on the right. Nucleotides whose aromatic protons undergo chemical shift variations upon the addition of two equivalents of **1a** are highlighted in orange and red (Δδ ≥0.04 and 0.08 ppm, respectively). Nucleotides with overlapped aromatic resonances are black-coloured, and residues whose aromatic signals were not affected by ligand binding are coloured grey. Solution conditions: 10 mM sodium phosphate pH 6.0 and 0.1 mM EDTA. (**C**) Model of a 1:1 complex between RRE loop IIB and **1a** (depicted with yellow carbon atoms), obtained from unrestrained docking calculations with the 4PMI PDB structure^12^. The image was generated with MOE 2019.0102 (www.chemcomp.com) and shows superimposed the converged docking poses of **1a**.

We next determined whether the terphenyl molecules blocked the interaction between Rev_34–50_ and subdomain IIB by binding to the RNA and, if so, whether they recognized subdomain IIB in a manner similar to the Rev_34–50_ α-helix. We first evaluated subdomain IIB association by measuring changes in the fluorescence intensity of a IIB_h_ RNA hairpin construct containing a fluorescein probe attached to unpaired loop IIB residue U23 (Fig. 1D)^21,22^. The binding curves obtained at low ionic strength indicated that all molecules associated to RRE subdomain IIB RNA, and were best fit with a two-site model (Figs. 4A and S4). However, there were significant differences among the compounds.

The two terphenyl molecules that inhibited the RRE subdomain IIB-Rev_34–50_ and full-length RRE-Rev interactions, **1a** and **1d**, presented lower second-site equilibrium dissociation constant (K_d_2) when compared to the other molecules, in addition to a low K_d_1. In contrast, compounds **1b**, **1c**, **2a** and **2b** had similarly low K_d_1 but higher K_d_2 values. On the other hand, terphenyls **3a**-**c** and **4**, bearing just three 2-aminoethyl groups, had both higher K_d_1 and K_d_2 constants (Fig. 4A and Tables 2 and S1). Experiments carried out at higher ionic strength supported these conclusions: the IIB_h_ K_d_’s of terphenyls **1a** and **1d** were considerably lower than those of compounds **3c** and **1b** (Fig. S5 and Table S2), both of which displayed weaker RRE-Rev inhibitory activity.

**Table 2:** IIB_h_ interaction parameters for selected 1,4-terphenyl molecules, measured by fluorescence intensity experiments.

cpd^a^	K_d_ (IIB_h_) (μM)	K_d_^b^ (IIB_h_+tRNA) (μM)	IIB-tRNA spec.^c^	K_d_^b^ (IIB_h_+LTR_d_) (μM)	IIB-DNA spec.^c^	K_d_ (TAR_h_) (μM)	${\frac{{{\boldsymbol{K}}}_{{\boldsymbol{d}}}({\boldsymbol{T}}{\boldsymbol{A}}{{\boldsymbol{R}}}_{{\boldsymbol{h}}})}{{{\boldsymbol{K}}}_{{\boldsymbol{d}}}({\boldsymbol{I}}{\boldsymbol{I}}{{\boldsymbol{B}}}_{{\boldsymbol{h}}})}}^{{\boldsymbol{c}}}$
**1a**	0.98 [5.9] (0.45–2.6 [2.6–11], 0.9785)	28 (17–51, 0.9634)	0.035	2.4 (1.0–6.9, 0.9523)	0.41	12 (9.6–14, 0.9868)	12
**1d**	0.87 [3.5] (0.68–1.1 [2.7–4.4], 0.9306)	7.8 (4.8–12, 0.9604)	0.11	2.2 (1.3–4.0, 0.9701)	0.40	22 [22] (18–29 [12–37], 0.9983)	25
**3c**	6.5 [26] (5.5–7.7 [22–31], 0.9825)	5.2 (3.5–7.6, 0.9766)	1.3	8.1 (4.1–17, 0.9508)	0.80	78 (51–99, 0.9510)	12
**1b**	0.51 [21] (0.34–0.77 [14–30], 0.9911)	8.7 (4.7–16, 0.9590)	0.059	6.5 (4.2–10, 0.9806)	0.078	>50	n/d

^a^For each compound (cpd), the table report best-fit IIB_h_ and TAR_h_ equilibrium dissociation constants (K_d_) obtained in 10 mM sodium phosphate (pH 6.6) and 0.1 mM EDTA. For binding curves best fit with a two-site model, first-site (K_d_1) and second-site (K_d_2; in brackets) dissociation constants are shown. For IIB_h_, K_d_’s were measured in the absence (IIB_h_) and presence of tRNA^Lys^ (IIB_h_+_t_RNA) or DNA duplex LTR_d_ (IIB_h_ + LTR_d_). 95% confidence intervals and R^2^ coefficients are shown in parentheses. n/d: not determined.

^b^Because of the ligand concentration range used in the experiments, these curves were fit with a one-site model and the corresponding K_d_ values should be considered approximate.

^c^The IIB-_t_RNA and IIB-DNA specificities of the interaction were quantified with the ratios K_d_(IIB_h_)/K_d_(IIB_h_ + _t_RNA) and K_d_(IIB_h_)/K_d_(IIB_h_ + LTR_d_), respectively. Interactions with specificity ratios close to 1 are specific, whereas those with ratios «1 are unspecific. IIB-TAR specificities were quantified with the ratio K_d_(TAR_h_)/K_d_(IIB_h_). When involving K_d_1 and K_d_2 values, the specificity ratios were calculated using the higher-affinity K_d_1 values.

To evaluate the specificity of subdomain IIB recognition, we measured binding to a control TAR_h_ hairpin containing the HIV-1 Tat-binding UCU bulge^23^ (Fig. 1D). The K_d_(TAR_h_)/K_d_(IIB_h_) specificity ratios of compounds **1a** and **1d** ranged between 3 and 25, depending on ionic strength (Figs. 4A, S4 and S5 and Tables 2 and S2). We further assessed specificity by duplicating the IIB_h_-23fl association experiments in the presence of a 10-fold molar excess of tRNA^Lys^ or a 26-base pair LTR_d_ DNA duplex^21^. The binding curves of the compounds were affected by the presence of competitive tRNA^Lys^ and to a lesser extent by LTR_d_ (Figs. 4A, S4 and S5 and Tables 2 and S2).

We next used NMR spectroscopy to identify the binding site of terphenyls **1a**, **1d** and **3c** within hairpin IIB_h_. All three compounds induced significant chemical shift changes in internal loop IIB and adjacent nucleotides only, and these variations were observed at low RNA:terphenyl molar ratios (1:1 and 1:2; Figs. 4B and S6). This indicated that the compounds interacted with the intended Rev_34–50_ binding site in subdomain IIB, and that the interactions were loop IIB-specific within the IIB_h_ hairpin. Under conditions of fast exchange between bound and unbound states, terphenyls **1a** and **1d** induced larger chemical shift perturbations than **3c** at the same molar ratios (Figs. 4B and S6). Assuming similar structures for the RNA-terphenyl complexes, this suggested that **1a** and **1d** had greater affinity for loop IIB relative to **3c**, in line with the results obtained with fluorescence experiments. We also detected intermolecular NOEs between H1’ of the extrahelical A19 loop nucleotide and the aminoethyl protons of compound **1a**, implying that the ligand associated to the loop from the major groove side, as observed for Rev_34–50_ (Fig. 1C)^11,12^. In fact, unrestrained docking calculations supported that one molecule of **1a** bound diagonally across the major groove of loop IIB, occupying the binding site of the N-terminal segment of the Rev_34–50_ helix (Fig. 4C). One pair of bilateral aminoethyl groups contacted phosphate groups located in opposite strands, whereas the other pair of aminoethyl groups bound to the pocket formed by the S-turn residues G21 and G22, and the extrahelical A19 nucleotide, where several phosphate groups are in close proximity to each other.

### Antiretroviral activity and cellular toxicity

**Figure 5:**
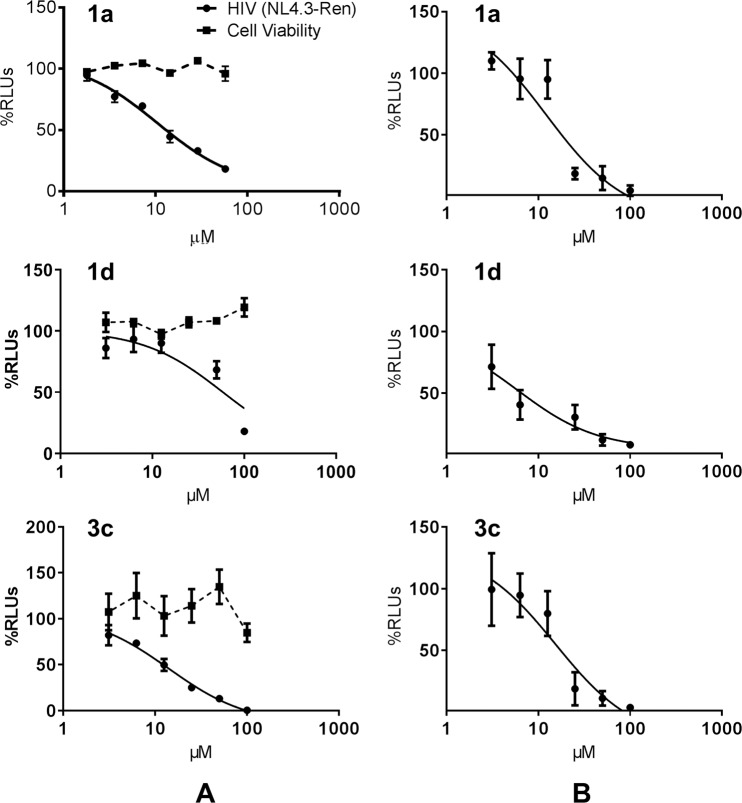
Antiviral activity and cellular toxicity of selected 1,4-terphenyl molecules **1a**, **1d** and **3c**. (**A**) Antiviral activity in HIV-1 infection assays and cellular toxicity as function of compound concentration. (**B**) Inhibition of post-integration steps in HIV-1 transfection assays. In all cases, the results are expressed as percentage of relative luminescence units (RLUs), where 100% is the level of viral replication obtained in the presence of the vehicle used to dissolve the compounds.

When the antiviral activities of 1,4-terphenyl compounds were evaluated with a cellular HIV-1 infection assay, the groups attached to the terphenyl scaffold were found to have a substantial impact on the activities, as observed in the previous *in vitro* experiments. Compounds **1a** and **3c** had significant activity in the infection experiment, with EC_50_ values of 10.6 and 14.1 μM respectively, followed by **3a** and **1d** (EC_50_ = 35.5 and 57.9 μM respectively), and finally **4** (Table 3 and Figs. 5A and S7). The remaining terphenyls were inactive at the assay concentrations (up to 100 μM). Notably, none of the terphenyl compounds were toxic at concentrations below 100 μM (Table 3 and Figs. 5A and S7). These experiments clearly show the antiviral effect of these compounds on the HIV-1 cycle.

**Table 3:** Results of cellular assays for 1,4-terphenyl compounds. Inhibitory activity (EC_50_) in experiments based on infection with HIV virus and transfection with HIV- and LTR-dependent vectors, and cellular toxicity (CC_50_).

Compound^a^	EC_50_ infection HIV-1 (μM)	EC_50_ transfection HIV-1 (μM)	EC_50_ transfection HIV-1 LTR (μM)	EC_50_ transfection HTLV LTR (μM)	CC_50_ (μM)
**1a**	10.6 (5.55–20.2, 0.9532)	12.2 (5.40–25.5, 0.8597)	7.20 (3.70–13.1, 0.6652)	16.7 (3.67–87.9, 0.7222)	>100
**1b**	>100	n/d	n/d	n/d	>100
**1c**	>100	n/d	n/d	n/d	>100
**1d**	57.9 (29.5–114, 0.7791)	6.00 (3.30–10.9, 0.8275)	3.20 (0.950–6.82, 0.9513)	24.9 (10.8–59.4, 0.7312)	>100
**2a**	>100	n/d	n/d	n/d	>100
**2b**	>100	n/d	n/d	n/d	>100
**3a**	35.5 (7.39–840, 0.8834)	n/d	n/d	n/d	>100
**3b**	>100	n/d	n/d	n/d	>100
**3c**	14.1 (8.5–23.5, 0.9707)	15.4 (6.80–33.8, 0.8178)	3.32 (1.19–6.60, 0.9645)	16.4 (3.88–82.4, 0.7578)	>100
**4**	>50 < 100	n/d	n/d	n/d	>100

^a^Confidence intervals and R^2^ values are shown in parentheses when applicable; n/d: not determined.

### Antiretroviral mechanism

The processes involved in the antiretroviral action of terphenyls **1a**, **1d** and **3c** were studied with additional cellular assays. A possible effect on reverse transcription was assessed by measuring the levels of early and late HIV-1 reverse transcripts in the absence and presence of two terphenyl concentrations. None of the molecules interfered with the levels of late reverse DNA, although terphenyls **1a** and **3c** diminished the levels of early viral DNA sequences by 25%, suggesting that early reverse transcription copying over the LTR region could be affected by the presence of the compounds (Fig. S8). Overall, we only found a partial action of terphenyls on reverse transcription.

In an experiment based on transfecting a full-length competent HIV-1 vector, the EC_50_ values for compounds **1a**, **1d** and **3c** ranged between 6.0 and 15.4 μM, close to those obtained in the infection assay (Fig. 5B and Table 3). This result indicated that the molecules mainly acted on transcriptional or post-transcriptional steps of the virus cycle.

**Figure 6:**
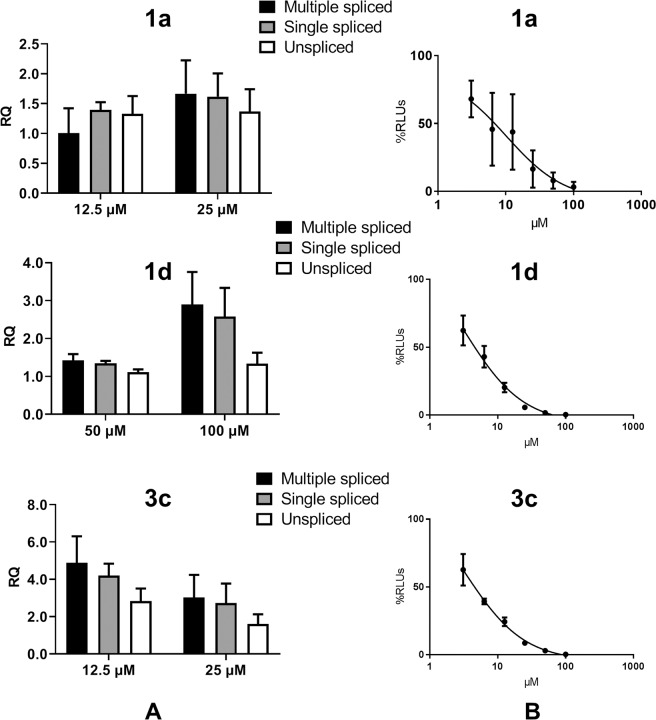
Effect of terphenyls **1a**, **1d** and **3c** on HIV-1 RNA splicing and LTR-dependent gene expression. (**A**) Relative quantities (RQ) of unspliced, single-spliced and multiple-spliced HIV-1 RNA transcripts in cells treated with two concentrations of **1a**, **1d** or **3c**. The image shows transcript quantities measured at 48 hours after infection, using transcript levels obtained from untreated cells as a reference (RQ = 1). (**B**) Inhibition of HIV-1 LTR-dependent gene expression as a function of terphenyl concentration. The results are expressed as a percentage of relative luminescence units (RLUs), where 100% is the luminescence level obtained in the presence of the vehicle used to dissolve the compounds.

We then specifically tested whether terphenyls **1a**, **1d** and **3c** had an effect on the RRE-Rev system in cell culture by quantifying the levels of unspliced, single-spliced and multiple-spliced viral transcripts using RT-qPCR experiments^21,22^. Given that splicing takes place in the nucleus, a blockage of the RRE-Rev system should reduce the levels of unspliced or single-spliced transcripts and increase the proportion of multiple-spliced species. Although we did not find statistically significant results, the clearest patterns consistent with RRE-Rev inhibition were detected for compound **1d** at 24 and 48 hours post-infection and 100 μM concentration (Figs. 6A and S9).

We also determined the effect of **1a**, **1d** and **3c** on viral transcription using an experiment based on transfecting a plasmid encoding a luciferase gene whose expression depends on the long terminal repeat (LTR) promoter of HIV-1^24^. All three compounds inhibited LTR-dependent expression with approximately similar EC_50_ values, which were slightly lower relative to those measured in the HIV-1 transfection experiments (Fig. 6B and Table 3). To further assess the mechanism of antiviral action of the compounds, we checked whether they also interfered with the regulatory sequences of HTLV-1, a closely related retrovirus. Our results showed inhibition of luciferase expression driven by the LTR promoter of HTLV-1 with higher EC_50_ values (Table 3).

The IIB_h_ association curves of terphenyls **1a** and **1d** were moderately displaced in the presence of a 10-fold molar excess of competitor LTR_d_ DNA duplex (Figs. 4A, S4 and S5 and Tables 2 and S2). This duplex comprised binding sequences of transcription factors NF-κB and Sp-1^21^, both of which have been shown to be essential for HIV-1 LTR promoter activity and virus replication^25–28^. To evaluate whether the observed effects of terphenyls **1a**, **1d** and **3c** on LTR-dependent expression were exerted through LTR association, we again used EMSA experiments to compare the association to a 58-base pair LTR_c_ sequence, corresponding to the core region of the HIV-1 LTR promoter and comprising several NF-κB and Sp1sites. The results indicated that terphenyls **1a** and **1d**, but not **3c** or the inactive control **1b**, associated to LTR_c_ at low μM concentrations (Fig. S10A). Isothermal titration calorimetry (ITC) experiments were subsequently applied to compare the binding of terphenyl **1a** to RRE subdomain IIB and a related DNA duplex. We obtained equilibrium dissociation constants of 0.5 and 6.3 μM, respectively, indicating that the affinity of the compound for RRE subdomain IIB was approximately one order of magnitude higher relative to the DNA duplex (Fig. S10B).

## Discussion

Here we describe the properties of terphenyl compounds with 1,4-bilateral substitutions designed to mimic all faces of the RNA-binding α-helix of Rev. By mimicking the Rev_34–50_ helix, the compounds were intended to occupy not only the high-affinity site in subdomain IIB of the RRE, but also the remaining RRE sites recognized by the Rev monomers, thereby blocking the formation of the RRE-Rev ribonucleoprotein complex, which remains an unexploited target in HIV-1 chemotherapy. Given the arginine-rich Rev_34–50_ α-helix, the terphenyl compounds contained several positively charged aminoalkyl side chains. The presence of this positive charge increased the aqueous solubility of the *p*-terphenyl scaffold, but compromised binding specificity and also influenced the antiviral mechanism of the compounds, as discussed below. No cellular toxicity, however, was detected for any of the compounds. Furthermore, a comparison between the active concentrations obtained in experiments involving purified nucleic acid and protein species and those carried out in cells (Tables 1, 2, 3 and S2) suggested optimal cellular and nuclear penetration properties.

Fluorescence anisotropy and electrophoresis experiments indicated that terphenyls **1a** and **1d**, containing four bilateral 2-aminoethyl side chains and two bilateral methyl groups on the central aromatic ring, inhibited formation of the RRE-Rev complex with low-μM IC_50_ values (Table 1 and Fig. 3). NMR spectroscopy analyses showed that these molecules occupied the Rev_34–50_ site in loop IIB (Figs. 4B and S6), and indicated that RNA recognition took place from the major groove, as reported for the helix (Figs. 1C and 4C)^11,12^.

Altogether, these results indicated that both **1a** and **1d** recognized RRE subdomain IIB and inhibited the RRE-Rev contact by mimicking Rev_34–50_. This finding is relevant because the majority of synthetic α-helix mimics match residues located on just one helical face^2–4^, and of the few multi-facial mimics described so far, none target a protein–RNA interaction^29,30^.

The curves of subdomain IIB-terphenyl association obtained with fluorescence intensity experiments at low ionic strength were best fit with a two-site model, and compounds **1a** and **1d** had low K_d_1 and K_d_2 values (Fig. 4A and Tables 2 and S1). A requirement of tight binding to both sites may explain why the remaining terphenyl compounds did not block the IIB-Rev_34–50_ or RRE-Rev interactions as efficiently (Table 1 and Figs. 3 and S3). Molecules with small structural changes relative to **1a** and **1d** (such as **1b**, **1c** or **2a-2b**) exhibited low K_d_1 but higher K_d_2 values, indicating that these changes affected terphenyl association to the second site. This could also explain why seemingly small structural differences make such an impact on the activity of these compounds; not only does the terphenyl have to bind well initially to the RRE subdomain IIB (K_d_1), but a second molecule must also bind to the RNA-ligand complex to exert significant inhibitory activity. This means that ligand-ligand interactions could be just as important as ligand-receptor interactions in this case, and high activity should be achieved with a balance of the two. On the other hand, compounds **3a**-**3c** and **4**, bearing three instead of four 2-aminoethyls, had both higher K_d_1 and K_d_2 constants (Fig. 4A and Table S1), indicating that four aminoalkyl chains are favoured for tighter binding to subdomain IIB. These RNA affinity differences between compounds were confirmed with fluorescence experiments carried out under higher ionic strength, closer to the ionic conditions present in a cell environment (Table S2 and Fig. S5).

The specificity ratios for subdomain IIB recognition relative to a control RNA molecule containing the TAR bulge ranged between 3 and 25 (Figs. 4A, S4 and S5 and Tables 2 and S2), similar to those obtained for previous 1,3-terphenyl molecules and the Rev_34–50_ helix itself^7^, and NMR spectroscopy titrations indicated specific binding to internal loop IIB (Figs. 4B and S6). However, the effect of competitive tRNA on the IIB association curves (Figs. 4A, S4 and S5 and Tables 2 and S2) indicated that due to the presence of positive charge, the specificity of these compounds was lower than that of RRE-Rev inhibitors identified by screening^21,22^ and other small-molecule RNA binders reported in the literature^31,32^.

When the viral effect of these compounds was assessed, a clear impact on HIV replication was found, with inhibitory concentrations in the micromolar range. Terphenyls **1a** and **1d**, but not **1b-c** or **2a-b**, inhibited HIV-1 replication with low-μM EC_50_ values (Figs. 5A and S7 and Table 3), suggesting that efficient RNA binding was important for the antiretroviral activity of terphenyl molecules containing four bilateral 2-aminoethyl groups. Transfection experiments involving a full-length provirus suggested that these molecules mainly acted on transcriptional and/or post-transcriptional processes of the viral cycle (Fig. 5B and Table 3). In agreement with this result, qPCR experiments measuring the effect of compounds **1a** and **1d** on the levels of reverse HIV-1 transcripts did not support a strong action on reverse transcription (Fig. S8). RT-qPCR experiments revealed a tendency of terphenyl **1d** to increase the levels of multiple-spliced HIV-1 transcripts relative to single-spliced and unspliced species, an effect consistent with cellular inhibition of Rev function (Figs. 6A and S9). In addition, **1a** and **1d** inhibited both HIV-1 and HTLV-1 LTR promoter-dependent gene expression (Fig. 6B and Table 3). The higher EC_50_ obtained for HTLV-1 relative to HIV-1 inhibition indicated that the compounds acted with some specificity on the HIV-1 LTR system. On the other hand, the inhibition of the HIV-1 LTR was likely based on transcriptional blockage, since increasing concentrations of **1a** and **1d** translated into decreased levels of the viral transcripts quantified in RT-qPCR experiments at 72 hours post-infection (Fig. S9). EMSA experiments analysing compound association to the core region of the HIV-1 LTR promoter revealed a perturbation of the DNA band at relatively low concentrations of terphenyls **1a** and **1d**, which was not detected for the inactive compound **1b** (Fig. S10A). This result supported a mechanism of transcriptional blockage based on LTR DNA association, which according to calorimetry experiments takes place with an affinity approximately 10 times smaller than that for RRE subdomain IIB association (Fig. S10B).

The terphenyl library included a subset of molecules containing three instead of four aminoethyl groups and among these, compounds **3a** and **3c** had significant antiretroviral activity (Table 3). Compounds **3a** and **3c** had reduced RNA affinity and RRE-Rev inhibition capacity relative to **1a** and **1d** (Figs. 3 and S3–S6 and Tables 1, 2 and S2), and terphenyl **3c** associated only very weakly to LTR DNA (Fig. S10A). This compound blocked LTR-dependent gene expression (Tables 3 and Fig. 6B). However, RT-qPCR experiments indicated that **3c** had a weaker effect on HIV-1 transcription relative to **1a** and **1d** (Figs. 6A and S9), suggesting a different mechanism of action relative to terphenyl molecules containing four aminoalkyl side-chains.

In conclusion, the results compiled in this manuscript indicate that four-arm terphenyls are multi-target agents acting on different steps of the HIV cycle, including transcription and Rev-dependent transport. Additional work will be needed to improve RRE binding affinity and specificity and to increase the antiretroviral activity of the terphenyl scaffold, like modifying the functional groups contained in the lateral chains, or inserting suitable asymmetric substituents in one or more of the benzene rings. Nevertheless, an encouraging property of all of these compounds is their reduced cellular toxicity relative to other antiretroviral RRE-Rev inhibitors^21,22^. A further potential advantage of these mimics may lie in the fact that the Rev_34–50_ motif is highly conserved among HIV isolates and serves not only as an RNA association element, but also as a non-canonical NLS and as a hotspot for interaction with cellular proteins^33–35^. This means that improved Rev_34–50_ mimics could possibly modulate other pathways in addition to RNA transport or splicing, increasing their chances of altering the viral cycle and making the emergence of target-related resistance more difficult. Multi-target compounds^36^ such as these terphenyl mimics are receiving increased attention in the drug discovery field and may be particularly effective for treating infectious and/or multi-factorial diseases such as AIDS, as they could reduce the likelihood of drug resistance and contribute to simplify current therapies.

## Methods

### Molecular modeling

The conformational space of the 1,4-terphenyl molecule **1a** was sampled using the MMFF94s force field^37^ and the Stochastic Search option of the MOE software package (CCG Inc.). The minimum-energy conformation was superposed on Rev_34–50_ -helices obtained from PDB structures 1ETG^11^, 4PMI^12^ and 3LPH^9^, to verify which side chains of the-helix were matched by the bilateral substituents of the terphenyl molecule. Three-dimensional models of a 1:1 complex of loop IIB with **1a** were built by docking the ligand into 1ETG and 1ETF^11^ as well as 4PMI^12^ RRE subdomain IIB structures using Gold 5.2^38^. For 4PMI, the missing atoms of the A19 base were added using standard geometries. In all cases, the binding site was defined with a large 20 Å radius around nucleotide C20, in agreement with the NMR chemical shift perturbations induced by the ligand. The calculations were unrestrained, employed the GoldScore fitness function^38^ and generated 20 solutions for each ligand with maximum search efficiency. For the 4PMI RNA structure, the docking run resulted in a converged set of eleven (55%) **1a** solutions that had pair-wise root mean square deviations lower than 1.64 Å and included all better-scored poses. We obtained similar results when docking **1a** into 1ETG or 1ETF structures.

### General methods for the synthesis of 1,4-substituted terphenyl compounds

Reactions were carried out under an inert atmosphere of N_2_ or Ar using standard Schlenk techniques or sealed tubes, unless otherwise indicated. Solvents were purified prior to use: THF was distilled from sodium and benzophenone and dichloromethane from CaCl_2_. Reagents were used as supplied by the commercial sources without further purification. The reactions were monitored by TLC using 0.25 mm precoated silica-gel plates. Visualization of the TLC plates was carried out with UV light and/or aqueous ceric ammonium molybdate solution or potassium permanganate stain. Flash column chromatography was performed with the indicated solvents on silica gel 60 (particle size: 0.040–0.063 mm). ^1^H, ^13^C and ^19^F NMR spectra were recorded with a Bruker 300 MHz spectrometer. A QTOF mass analyser system was used for HRMS measurements. Stock solutions were prepared by dissolving each compound in H_2_O at a concentration of 5 mM. The concentration of all terphenyl stocks was verified by NMR spectroscopy using the ERETIC utility of Topspin 3.5 (Bruker Biospin).

### RNA, DNA, peptide and protein samples

The composition and preparation of the following species have been described in detail in previous reports^7,21^: 28-nt subdomain IIB RNA oligonucleotides IIB_h_ and IIB_h_-23fl (Fig. 1D), 234-nt RNA sequence RRE (Fig. 3B), 26-nt and 16-nt self-complementary DNA oligonucleotides LTR_d_ and DNA_d_, full-length protein Rev, unlabelled Rev_34–50_ peptide revp, and fluorescein isothiocyanate (FITC)-labelled Rev_34–50_ peptide frevp. Additionally, a TAR_h_-8fl RNA molecule containing a FITC probe linked to extra-helical loop nucleotide U8 (Fig. 1D) was purchased HPLC-purified from Horizon Discovery and desalted; a 58-base pair DNA duplex corresponding to the core region of the HIV-1 LTR promoter (LTR_c_), was obtained by PCR amplification from a HIV-1 LTR-luc plasmid^24^ utilizing GGGACTTTCCGCTGGGGAC (forward) and GGCGGGACTGGGGAGTGGC (reverse) primers; and *Escherichia coli* tRNA^Lys^ was transcribed *in vitro* from a BstNI-digested pUC19 plasmid and purified by gel electrophoresis. RRE, Rev and LTR_c_ were used in electrophoretic mobility shift assays (EMSA), and DNA_d_ was employed as a specificity control in these experiments. Unlabelled IIB_h_ was utilized in nuclear magnetic resonance (NMR) spectroscopy and fluorescence anisotropy experiments. IIB_h_-23fl and TAR_h_-8fl were employed in fluorescence intensity experiments, and tRNA^Lys^ and LTR_d_ were used as RNA and DNA specificity controls in the fluorescence intensity tests.

### Fluorescence anisotropy

These experiments were conducted in a Victor X5 (PerkinElmer) plate reader as described before^7,21^, using 10 nM frevp and 60 nM IIB_h_. Each experiment had one positive (a mixture of IIB_h_ and frevp, equivalent to 0% inhibition) and two negative (isolated frevp as well as a mixture of IIB_h_, frevp and neomycin B) controls. Since the fluorescence of several 1,4-terphenyl compounds was found to interfere with this assay at high concentrations, a baseline correction was performed: anisotropy data of all isolated molecules were generated and subtracted from the signal obtained in the presence of IIB_h_/frevp at the same concentration values. IC_50_ values were then calculated with GraphPad Prism using the following sigmoidal inhibitory model:

$$A=\frac{100}{1+{10}^{\log C-\log I{C}_{50}}}$$

where A is normalized anisotropy and C is total concentration of compound. We only quantified with this equation the activity of those compounds that, according to the experiment controls, induced the expected reduction in anisotropy after baseline correction; all other molecules were considered inactive. Each fluorescence anisotropy experiment was repeated at least two times.

### Electrophoretic mobility shift assays (EMSA)

The experiments monitoring RRE-Rev inhibition utilized 78 nM full-length RRE and 1.32 μM full-length Rev dissolved in 10 mM HEPES pH 7.5, 300 mM KCl, 1 mM MgCl_2_ and 0.5 mM EDTA binding buffer, and increasing concentrations of each compound up to 50 μM^21,39^. The reactions were incubated at room temperature for 20 minutes and loaded onto 8% polyacrylamide gels with TB running buffer. Gels were run at 4 °C for 1–4 hours at 150 V, and the bands were stained with SYBR gold and quantified with Quantity One 4.1 analysis software. Experiments monitoring binding to the 58-base pair HIV-1 LTR_c_ core segment utilized 20 nM LTR_c_ duplex dissolved in the same binding buffer, and increasing concentrations of each compound. The reactions were similarly incubated and loaded onto 20% polyacrylamide gels with TB running buffer. The gels were run at 4 °C for 3.5 hours at 150 V, and the bands were stained and quantified as described above. The specificity of LTR_c_ association was evaluated by duplicating the experiments in the presence of a 100-fold molar excess of DNA_d_ duplex (28-fold base-pair molar excess). In all cases, we monitored the disappearance of the band corresponding to high-order RRE-Rev complexes or free LTR_c_, and 50% response RC_50_ values were determined with Prism by fitting the data to a sigmoidal inhibitory model:

$$I-{I}_{min}=\frac{{{\rm{I}}}_{max}C}{1+{\rm{C}}/R{C}_{50}}$$

where *I* is the intensity of the band corresponding to LTR_c_ or high-order RRE-Rev species at compound concentration *C*, *I*_*max*_ the best-fit value for maximum intensity, and *I*_*min*_ the minimum intensity obtained at the highest concentration of inhibitor. All EMSA experiments were repeated three times for each compound.

### Fluorescence intensity

These experiments measured association to IIB_h_-23fl or TAR_h_-8fl RNA molecules labelled with fluorescein at extrahelical loop nucleotides U23 and U8, respectively (Fig. 1D), and were carried out under two different ionic conditions in a Victor X5 plate reader, using excitation and emission wavelengths of 485 and 520 nm, respectively. We also attempted to measure association to an alternative IIB_h_ hairpin containing 2-aminopurine instead of adenine at unpaired loop IIB residue A19^21^, but all terphenyls fluoresced at the excitation wavelength of this fluorophore. IIB_h_-23fl or TAR_h_-8fl (at 100 nM concentration) was equilibrated for 5 minutes after each ligand addition in a buffer containing either 10 mM sodium phosphate pH 6.6 and 0.1 mM EDTA or 10 mM HEPES pH 7.5, 200 mM KCl and 2 mM MgCl_2_. In addition to the TAR_h_ specificity control, the RNA and DNA specificity of the IIB_h_ interactions was assessed by duplicating the experiments in the presence of a 10-fold molar excess (1 μM) of either tRNA^Lys^ or DNA duplex LTR_d_. The equilibrium dissociation constants K_d_ were determined by fitting the fluorescence intensity curves with DYNAFIT^40^. We used one-site, two independent-sites and two interacting-sites binding models for all curves, and the best model was automatically selected by model discrimination analysis^40^, except where indicated. The final graphs were plotted with Prism. All fluorescence intensity experiments were performed at least two times for each compound and condition.

### NMR spectroscopy

NMR spectra were acquired in a Bruker Avance III 500 MHz or cryoprobe-equipped Bruker Avance 600 MHz spectrometers, and analysed using Topspin 1.3 (Bruker Biospin) and Sparky 3.110^41^. The IIB_h_ RNA samples were previously microdialyzed in an aqueous solution containing 10 mM sodium phosphate (pH 6.0) and 0.1 mM EDTA. The interaction of 30–50 μM (5–7 ODs) IIB_h,_ samples with terphenyl compounds was monitored at 27 °C using one- and two-dimensional (TOCSY) experiments at increasing ligand:RNA molar ratios: 1:1, 2:1, and 4:1. The complex of IIB_h_ with **1a** was also analysed at 2:1 and 4:1 **1a**:RNA ratios with NOESY experiments employing a recycle delay of 2 seconds and 600 or 800 ms mixing time.

### Isothermal titration calorimetry

These experiments were performed at 25 °C in MicroCal PEAQ-ITC or Nano-ITC microcalorimeters, and the data was subsequently analysed with MicroCal or Nanoanalyze software, respectively. All species were dissolved in aqueous solutions containing 10 mM sodium phosphate (pH 7.4 or 8.2) and 0.1 mM EDTA. For the IIB_h_:**1a** interaction the pH was 7.4, and 10 or 20 μM solutions of IIB_h_ in the sample cell were titrated with 19 injections of 350 or 500 μM solutions of **1a**. We previously reported that titrations of IIB_h_ with 1,3-terphenyl compounds exhibited a higher affinity transition followed by a complex, lower affinity step^7^. The IIB_h_:**1a** titration experiments focused on the higher affinity step, associated to loop IIB binding as revealed by NMR spectroscopy. For the DNA_d_:**1a** interaction, the pH was 8.2, and 800 or 900 μM solutions of **1a** were titrated into 20 μM solutions of DNA_d_ duplex in the sample cell with 19 injections of 2 µL. In both cases, the titration experiments were repeated three times, and the resulting association curves were fitted using a model with a single set of binding sites^42^.

### Plasmids, viruses and cells for *ex vivo* assays

Vectors pNL4.3-Luc and pNL4.3-Ren were generated by cloning the luciferase and renilla genes, respectively, in the nef site of HIV-1 proviral clone pNL4.3^43^. These constructs generate replication-competent viruses as previously shown^44^. Plasmids pLTR-luc^24^ and pLTR(HTLV)-luc^45^ carried a luciferase gene under the control of the HIV-1 or HTLV-1 LTR promoters, respectively. MT-2^46^ and 293 T cells were cultured as described previously^21^.

### Evaluation of anti-HIV-1 activity and cellular toxicity

The methodology used to perform and analyse these experiments has been reported elsewhere^7,21,22^. Briefly, infectious supernatants were obtained from transfection of plasmid pNL4.3-Ren on 293 T cells, MT-2 cells were infected with these supernatants in the presence of the compounds, and anti-HIV activity quantification was performed 48 h post-infection by determining luciferase activity in cell lysates compared to a non-treated control (100%). Cellular viability was evaluated in mock infected cells similarly treated with the same concentrations of compounds using the CellTiterGlo (Promega) assay. 50% effective (EC_50_) and cytotoxic (CC_50_) concentrations were calculated with Prism using log(inhibitor) vs response non-linear regression analyses. The results represent the average of at least three independent experiments.

### Quantification of early and late reverse transcription

MT-2 cells were pre-treated with two different concentrations of terphenyl molecules **1a**, **1d** or **3c**, selected on the basis of the observed RRE-Rev IC_50_ and cellular EC_50_ values, and infected with NL4.3 wild-type HIV-1 for 5 hours. Total genomic DNA was isolated with a QIAamp DNA blood mini kit (Qiagen) and quantified by spectrophotometry. Early and late viral DNAs were quantified by qPCR as previously described^47^. qPCR was performed in triplicate in a StepOne Real-Time PCR system using standard cycling conditions. Serial dilutions of genomic DNA from the 8E5 cell line, which contain a single integrated copy of HIV-1^43^, were used as standard curve. The CCR5 gene was used as an endogenous control.

### Transfection assays

MT-2 cells were transfected with plasmids containing a luciferase reporter gene whose expression was under the control of the full length proviral HIV-1 (NL4.3-luc), the HIV-1 LTR promoter (pLTR-luc), or the HTLV-1 LTR promoter (pLTR(HTLV)-luc). After transfection, cells were treated with different compound concentrations, and activity quantification was performed 48 h later by determining luciferase activity in cell lysates as described^7,21,22^. 50% effective (EC_50_) concentrations were calculated with Prism using log(inhibitor) vs response non-linear regression analyses, and the results represent the average of at least three independent experiments.

### Analysis of HIV-1 RNA splicing

MT-2 cells were infected with a NL4.3 virus for 2 hours (10 ng/10^6^ cells) and treated with two different concentrations of compounds **1a**, **1d** or **3c** for 24, 48, 72 or 96 hours. The compound concentrations were chosen on the basis of the observed RRE-Rev IC_50_ and cellular EC_50_ values. Total cellular RNA was isolated, treated with DNase I and reverse-transcribed as previously described^21,22^. Unspliced, single-spliced and multiple-spliced HIV-1 RNA transcripts were quantified by qPCR relative to a control obtained from untreated cells, using the primers described by Mohammadi *et al*.^48^, and GAPDH as an endogenous control.

## Supplementary information

Supplementary Information.
