# Supplementary material for: Nucleic acid recognition and antiviral activity of 1,4-substituted terphenyl compounds mimicking all faces of the HIV-1 Rev protein positively-charged α-helix
Source: Sci Rep. 2020 Apr 28;10:7190. doi: 10.1038/s41598-020-64120-2 (PMC7188855; doi:10.1038/s41598-020-64120-2)
Supplement: Supplementary file 1 — Supplementary Information. [file 41598_2020_64120_MOESM1_ESM.pdf]

Nucleic acid recognition and antiviral activity of 1,4-substituted terphenyl compounds mimicking all faces of the HIV-1 Rev protein positively-charged α-helix

Cristina Medina-Trillo, Daniel M. Sedgwick, Lidia Herrera, Manuela Beltrán, Ángela Moreno, Pablo Barrio, Luis. M. Bedoya, José Alcamí, Santos Fustero and José Gallego

Supporting Information

1. Supplementary Figures S1-S11 (pages s1-s11)
2. Supplementary Tables S1-S2 (pages s12-s13)
3. Synthesis and characterization of 1,4-terphenyls (pages s14-s32)
4. References (page s33)

1. Supplementary Figures

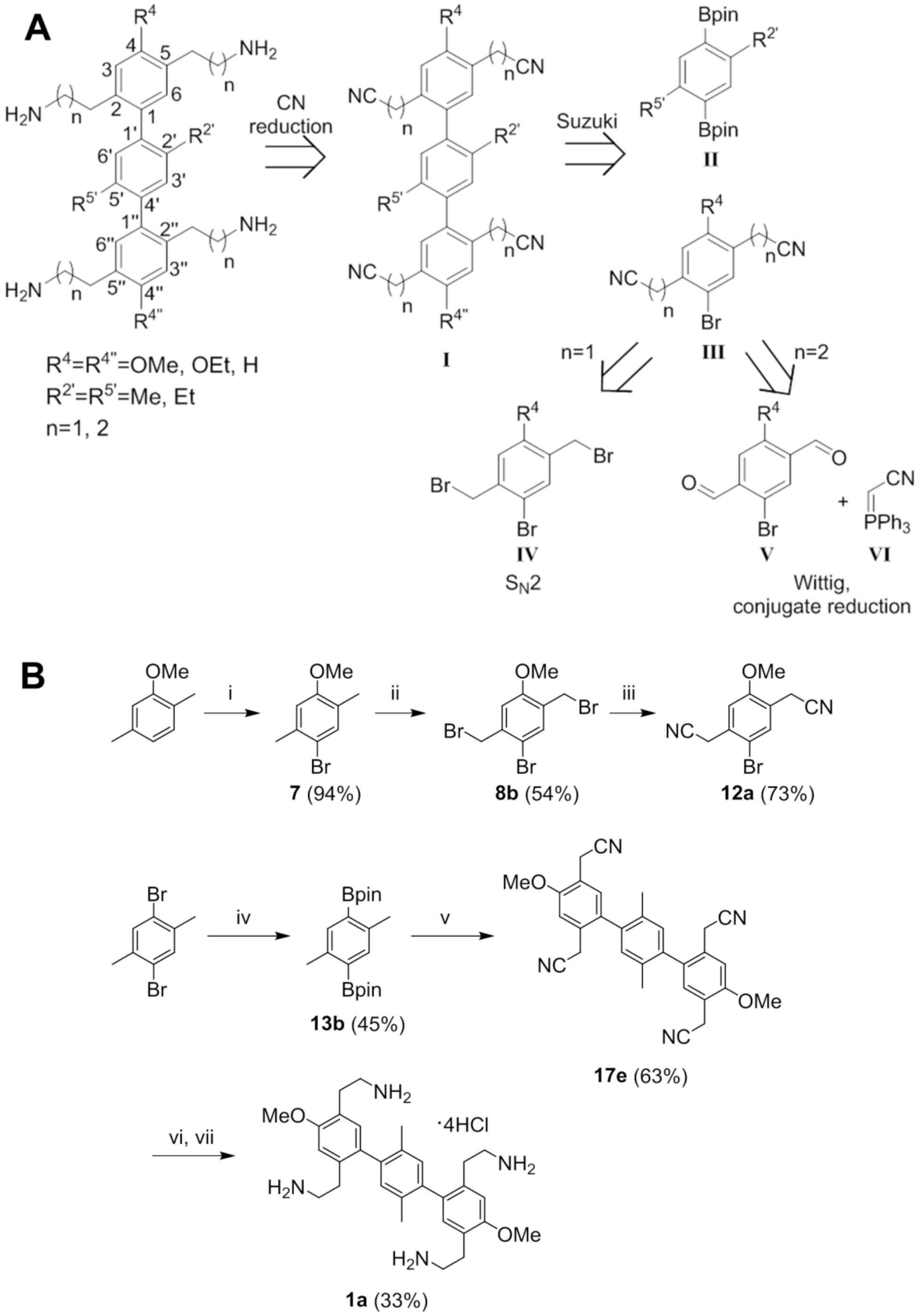

Reagents and conditions: i) Br_2, CCl_4, 2h. ii) NBS, $(PhCO_2)_2$, CCl_4, 90ºC, 3h. iii) NaCN, 18-corona-6, KI, MeCN:H_2O (8:1), 20h. iv) B_2pin_2, KOAc, $PdCl_2$(dppf), 1,4-Dioxane, 120ºC, 1h (microwave oven). v) **12a**, K_3PO_4, $Pd(OAc)_2$, PPh_3, MeCN:H_2O (7:3), 120ºC, 1h (microwave oven). vi) BH_3-THF, 85ºC, 5 days. vii) HCl, H_2O, 70ºC, 2h. viii) HCl (2M in Et_2O), MeOH, 1h.

Figure S1. Synthetic route and chemical structure of 1,4-substituted *p*-terphenyl compounds. (**A**) Retrosynthesis of 1,4-substituted *p*-terphenyl compounds, highlighting the key synthons necessary to achieve 1,4-terphenyls with diverse groups at $R^{2'}$, $R^{5'}$, R^4 and $R^{4''}$, as well as differing alkylamine chain lengths. (**B**) Example synthesis of terphenyl **1a**.

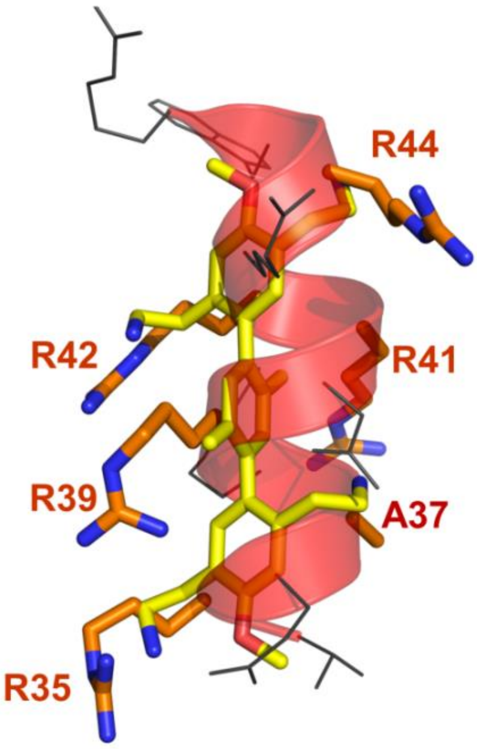

Figure S2. Mimicry of all faces of an α-helix by terphenyl compounds with 1,4-bilateral substituents. The minimum-energy conformation of terphenyl **1a** (with yellow carbon atoms) is shown superposed on the N-terminal $T_{34}RQARRNRRRRWR_{46}$ segment of the $Rev_{34\text{-}50}$ α-helix (red ribbon), obtained from PDB structure 1ETG.[1] Starting arbitrarily from $Rev_{34\text{-}50}$ residue R35, the bilateral substituents of the terphenyl molecule approximately match the side chains of R35 (i), A37 (i+2), R39 (i+4), R41 (i+6), R42 (i+7) and R44 (i+9), represented with orange carbons. The remaining Rev residues are depicted with thin black lines. The N-terminal $Rev_{34\text{-}46}$ segment contains most of the Rev residues that are essential for the interaction with subdomain IIB.[1-3] Its position when bound to loop IIB (Figure 1C) is matched by that of bilaterally-substituted terphenyl molecules in theoretical (Figure 4C) and NMR-supported[4] models of subdomain IIB-terphenyl complexes. The image was generated with MOE 2019.0102 (www.chemcomp.com).

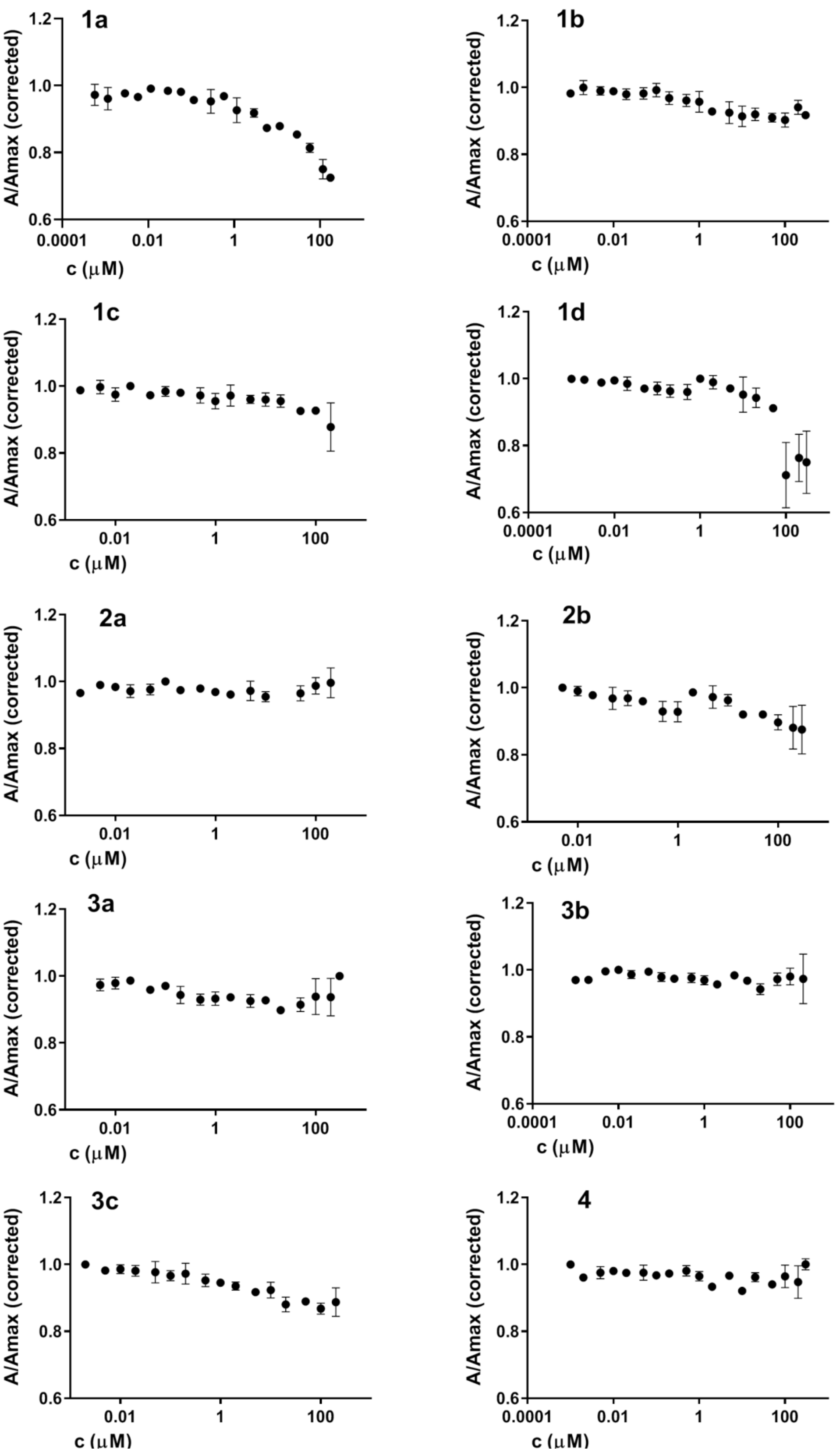

Figure S3. Fluorescence anisotropy experiments. Inhibition of the IIB_h-Rev_{34-50} interaction by 1,4-terphenyl compounds. The ratio A/A_{max} (where A is anisotropy and A_{max} is maximum anisotropy) is plotted against compound concentration c (logarithmic scale). The baselines were corrected as explained in Methods to minimize the fluorescence signal observed for several isolated 1,4-terphenyl compounds at high concentration. Ionic conditions: 100 mM KCl, 20 mM NaCl and 2 mM $MgCl_2$.

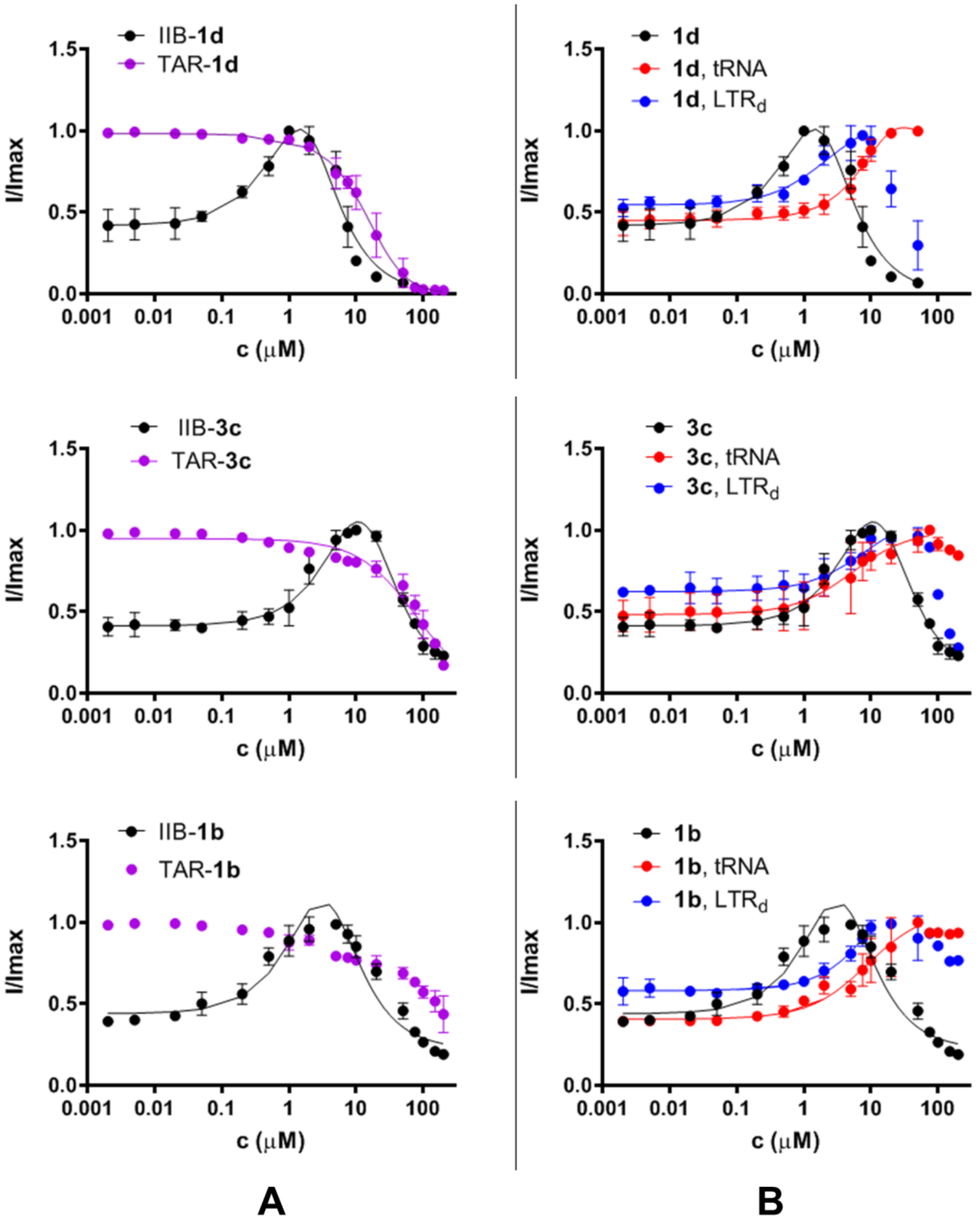

Figure S4. RRE subdomain IIB recognition by compounds **1d**, **3c** and **1b**, studied with fluorescence intensity experiments under low ionic strength conditions (10 mM sodium phosphate pH 6.6 and 0.1 mM EDTA). (**A**) Comparison of IIB_h binding curves (black circles) with TAR_h association curves (magenta circles). (**B**) Comparison of IIB_h association curves obtained in the absence (black circles) and presence of a 10-fold molar excess of unlabelled competitor RNA ($tRNA^{Lys}$; red circles) or unlabelled competitor double-helical DNA (LTR_d; blue circles).

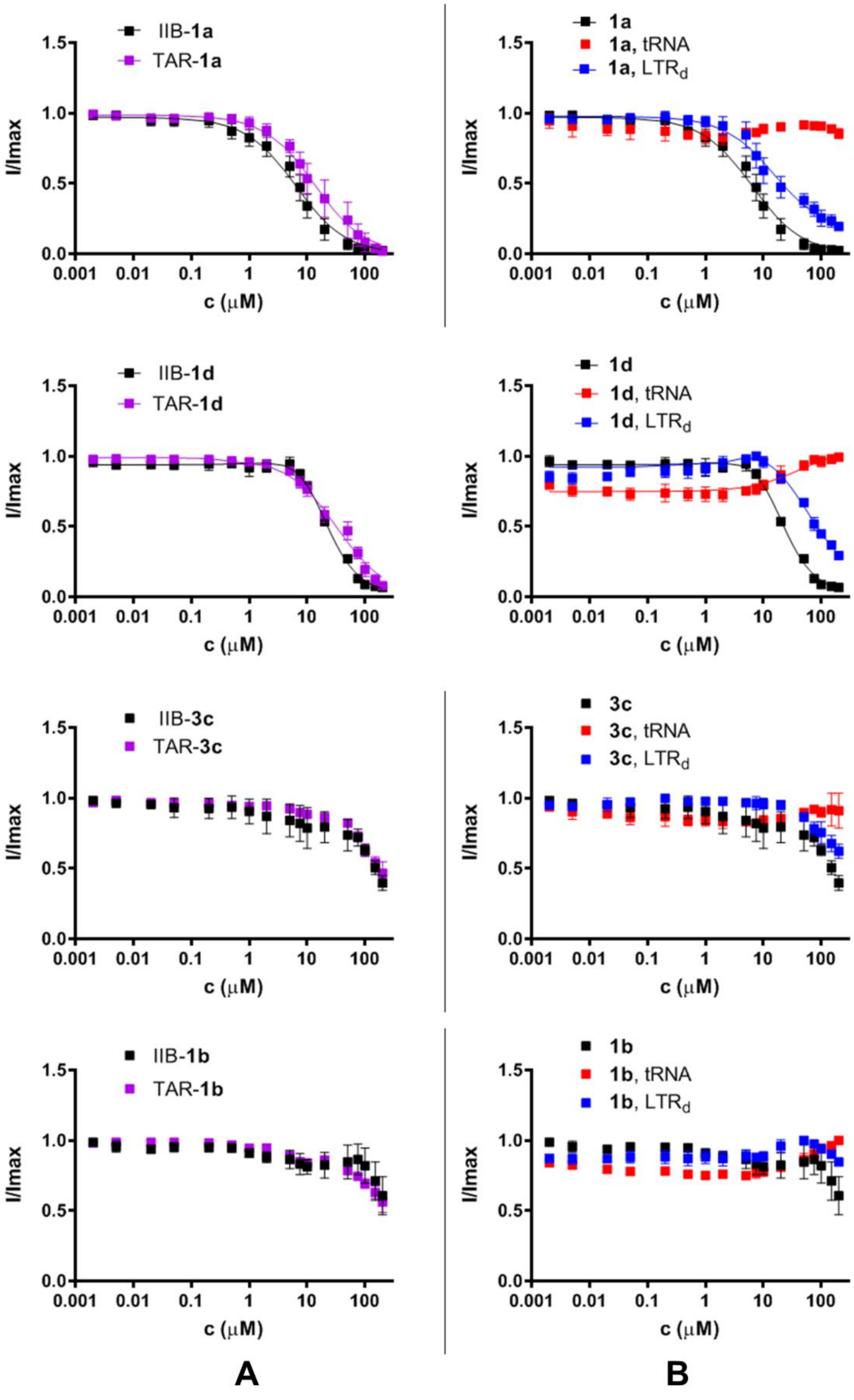

Figure S5. RRE subdomain IIB recognition by 1,4-terphenyl compounds **1a**, **1d**, **3c** and **1b**, studied with fluorescence intensity experiments under higher ionic strength conditions (10 mM HEPES pH 7.5, 200 mM KCl and 2 mM $MgCl_2$). (**A**) Comparison of IIB_h binding curves (black squares) with TAR_h association curves (magenta squares). (**B**) Comparison of IIB_h association curves obtained in the absence (black squares) and presence of a 10-fold molar excess of unlabelled competitor RNA ($tRNA^{Lys}$; red squares) or unlabelled competitor double-helical DNA (LTR_d; blue squares).

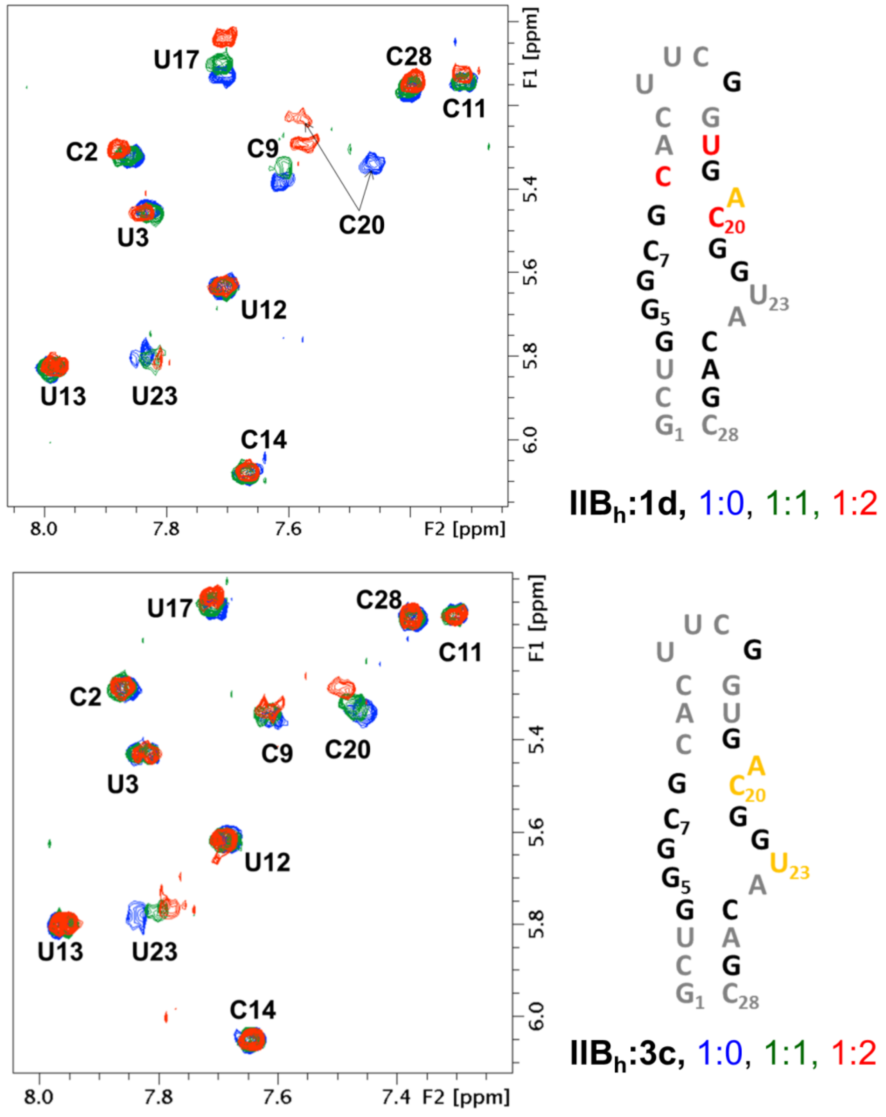

Figure S6. Titration of IIB$_h$ hairpin with terphenyls **1d** and **3c**, monitored by NMR spectroscopy. The H5-H6 region of the TOCSY spectrum of unbound IIB$_h$ (blue) is superposed on the spectra of complexes with increasing RNA:drug molar ratios, colour-coded as indicated in the graphs. A map of the binding sites of **1d** and **3c** in the IIB$_h$ hairpin is shown on the right of the spectra. Nucleotides whose aromatic protons undergo chemical shifts variations upon the addition of two equivalents of compound are highlighted in orange and red ($\Delta\delta \geq 0.04$ and 0.08 ppm, respectively). Nucleotides with overlapped aromatic resonances are black-coloured, and residues whose aromatic signals were not affected by ligand binding are coloured grey. Solution conditions: 10 mM sodium phosphate pH 6.0 and 0.1 mM EDTA.

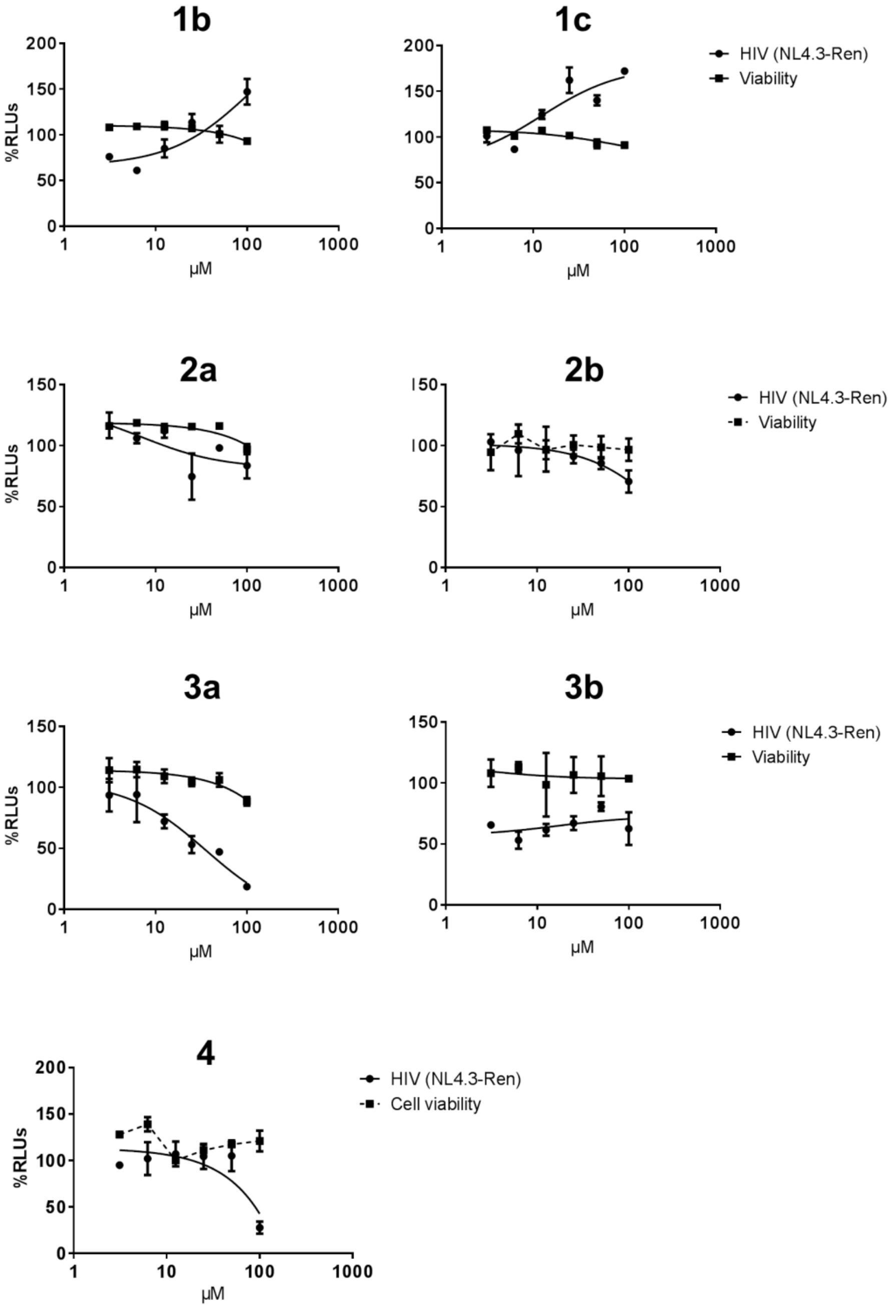

Figure S7. Antiviral activity in infection experiments (NL4.3-Ren) and cellular viability as a function of compound concentration for 1,4-terphenyls **1b**, **1c**, **2a**, **2b**, **3a**, **3b** and **4**. Results are expressed as percentage of RLUs, where 100% are MT-2 cell infections carried out with the vehicle used to dissolve the compounds.

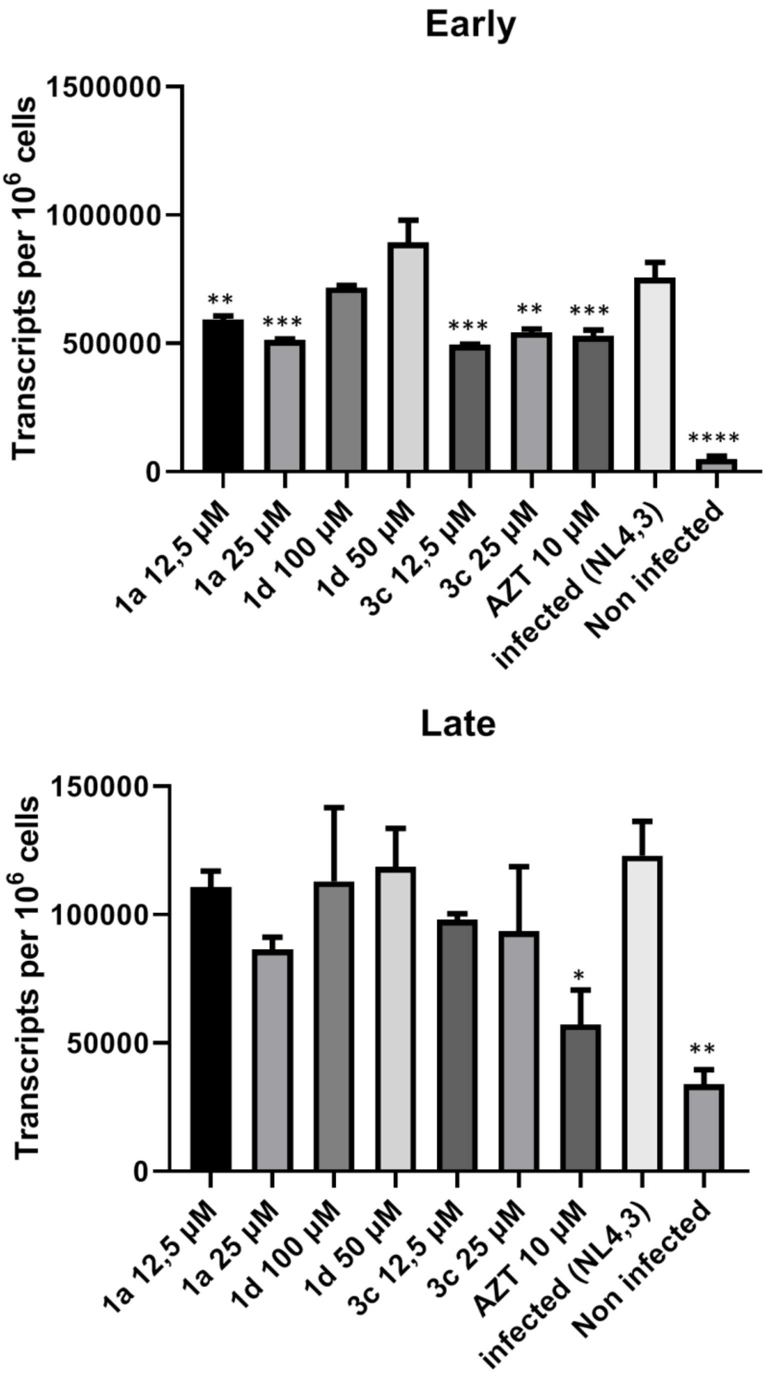

Figure S8. Effect of terphenyls **1a**, **1d** and **3c** on HIV-1 reverse transcription. Early and late HIV-1 DNAs were quantified by qPCR in HIV-1 (NL4.3)-infected MT-2 cells in the presence of two different concentrations of **1a**, **1d** or **3c,** or 10 μM of the reverse transcriptase inhibitor azidothymidine (AZT). Results are shown as number of early (upper panel) and late (lower panel) HIV-1 cell copies, using 8E5 cells as a reference for the number of HIV-1 copies in cell culture. NL4.3 (untreated HIV-1 infection) was used as a control. p values were determined with ANOVA analyses (* p <0.05, ** p <0.01, *** p <0.001, **** p<0.0001).

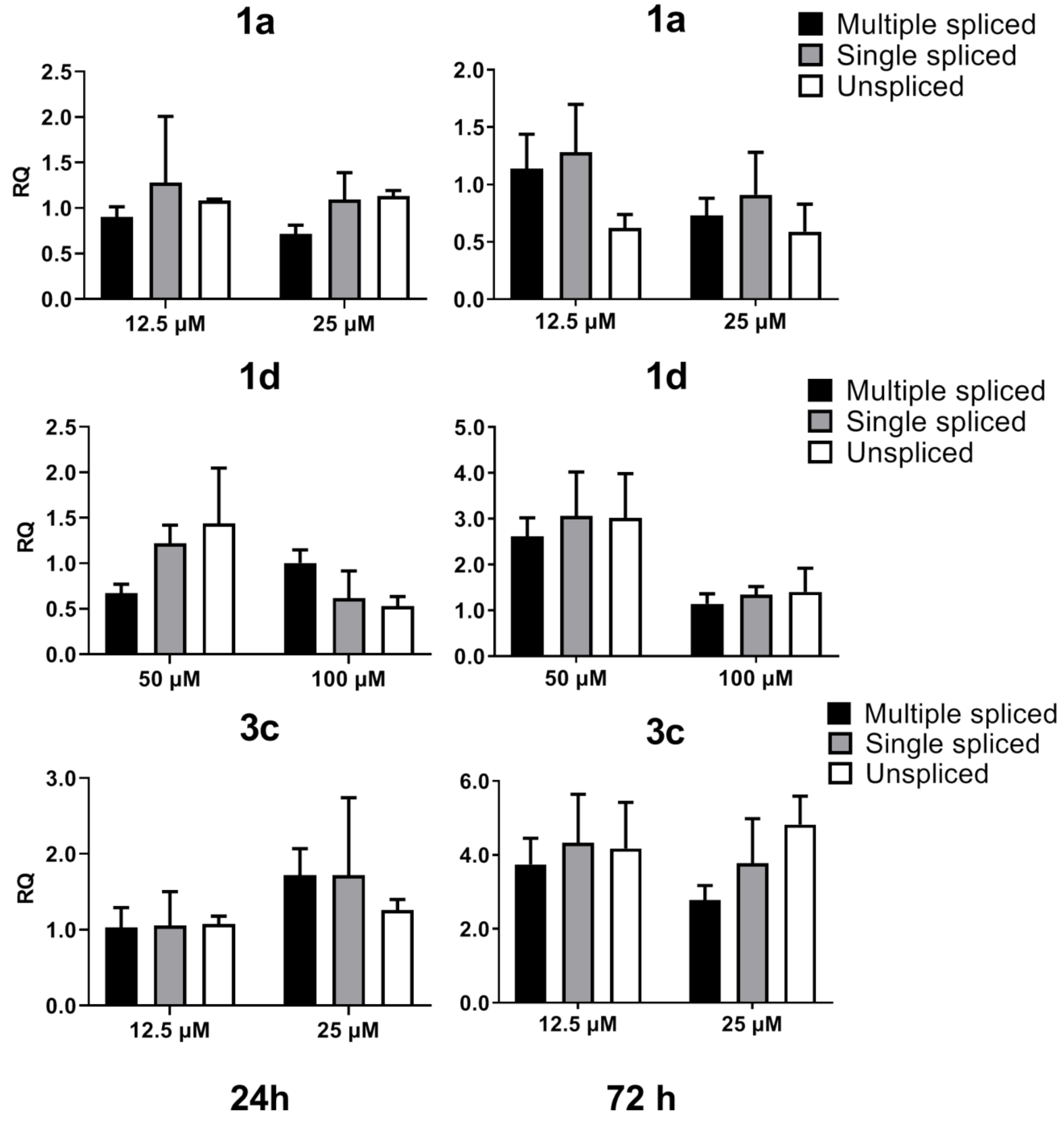

Figure S9. Effect of terphenyls **1a**, **1d** and **3c** on HIV-1 RNA splicing. The image shows relative quantities (RQ) obtained at 24 and 72 hours after infection of unspliced, single-spliced and multiple-spliced HIV-1 RNA transcripts in cells treated with two concentrations of terphenyls **1a**, **1d** or **3c**. RNA levels obtained from untreated cells were used as a reference (RQ=1).

A

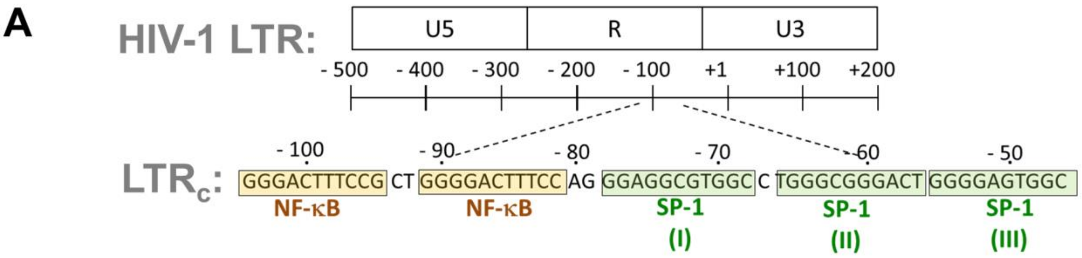

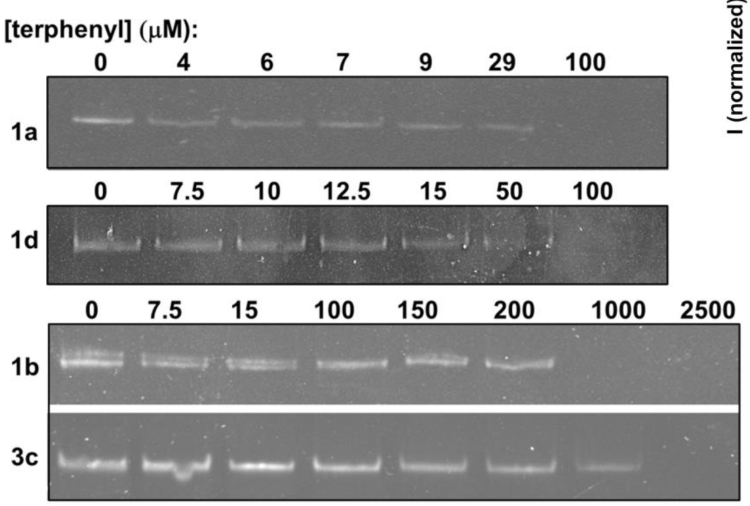

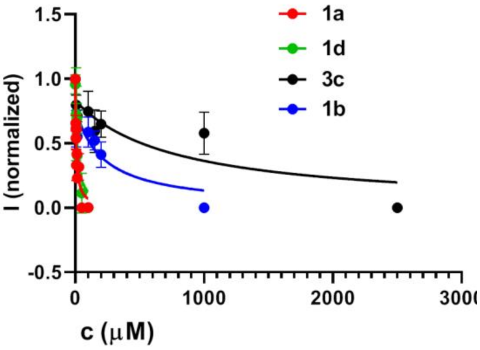

cpd	EC_{50} (μM)	EC_{50} (DNA_d, μM)
**1a**	7.7 (5.6-11, 0.9032)	7.7 (4.5-13, 0.7783)
**1d**	14 (7.9-29, 0.7184)	5.7 (3.1-11, 0.7653)
**3c**	779 (368-1666, 0.6917)	n/d
**1b**	210 (108-439, 0.7170)	n/d

B

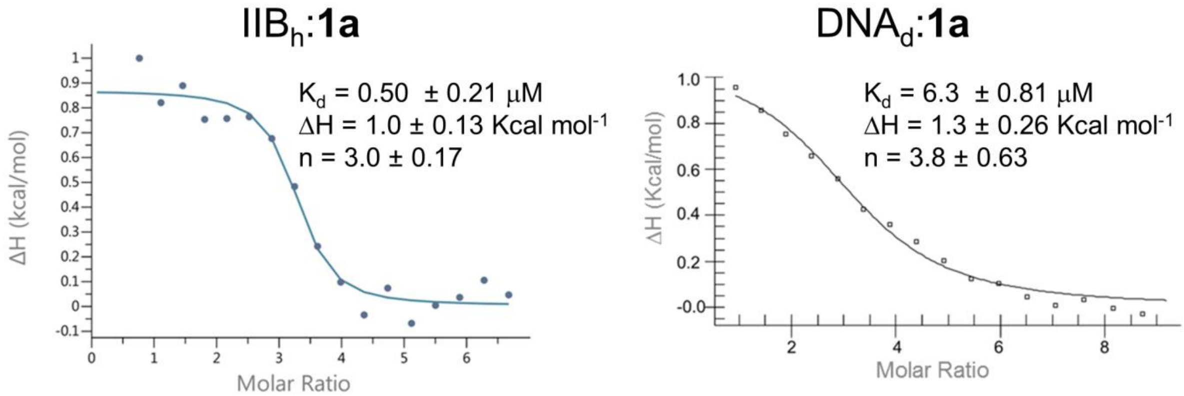

Figure S10. Association of terphenyl compounds to the core region of the HIV-1 LTR DNA promoter. (**A**) EMSA analysis of LTR_c binding. Top: scheme of the HIV-1 LTR promoter and LTR core sequence (LTR_c) used in the experiments, indicating the position of the binding sites of transcription factors NF-κB and Sp1. Bottom: EMSA experiments, showing plots of unbound LTR_c band intensity as a function of compound concentration, and 50% effective concentrations of terphenyls **1a**, **1d**, **3c** and **1b** for LTR_c association, measured with 20 nM LTR_c duplex in the absence and presence of a 100-fold molar excess (2 μM) of competitor DNA_d. The error bars represent the standard deviation of three independent experiments, and confidence intervals and R^2 values are shown in parentheses (n/d: not determined). Solution conditions: 10 mM HEPES pH 7.5, 300 mM KCl, 1 mM $MgCl_2$ and 0.5 mM EDTA. (**B**) Comparison of the binding affinities of terphenyl **1a** for RRE hairpin IIB_h and DNA duplex DNA_d, obtained with isothermal titration calorimetry experiments. The dissociation constants (K_d), binding enthalpies (ΔH) and stoichiometries (n) indicated on the graphs correspond to the average and standard deviation of three independent experiments. Solution conditions: 10 mM sodium phosphate (pH 7.4 or 8.2) and 0.1 mM EDTA.

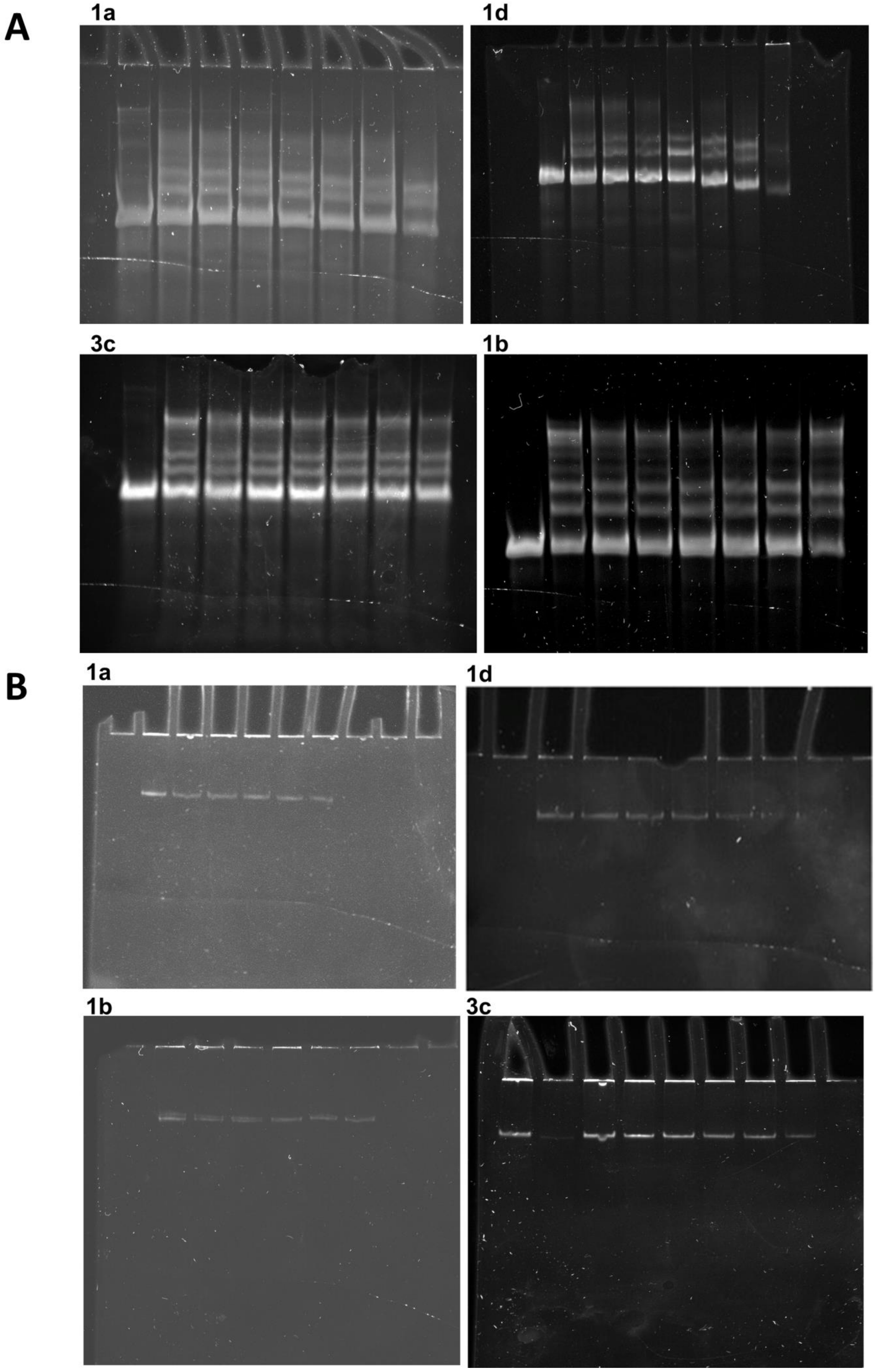

Figure S11. Unprocessed versions of the gel images contained in Figures 3B (**A**) and S10A (**B**).

2. Supplementary Tables

Table S1. Comparison between first-site and second-site equilibrium dissociation constants for the interaction between hairpin IIB_h and 1,4-terphenyl molecules, obtained with fluorescence intensity experiments under low ionic strength conditions.

Compound	$K_{d,1}$ (μM)	$K_{d,2}$ (μM)	R^2
**1a**	0.98 (0.45-2.6)	5.9 (2.6-11)	0.9875
**1b**	0.51 (0.34-0.77)	21 (14-30)	0.9911
**1c**	0.19 (0.12-0.31)	74 (53-88)	0.9854
**1d**	0.87 (0.68-1.1)	3.5 (2.7-4.4)	0.9306
**2a**	0.67 (0.46-0.99)	25 (18-35)	0.9907
**2b**	0.11 (0.063-0.19)	46 (30-71)	0.9825
**3a**	8.9 (7.7-10)	36 (31-42)	0.9751
**3b**	5.5 (4.7-6.4)	22 (19-26)	0.9838
**3c**	6.5 (5.5-7.7)	26 (22-31)	0.9825
**4**	3.7 (2.1-7.2)	33 (19-53)	0.9775

All IIB_h binding curves were best fit with a two-site model. The Table shows R^2 values and 95% confidence intervals (in parentheses). Solution conditions: 10 mM sodium phosphate pH 6.6 and 0.1 mM EDTA.

Table S2. IIB_h interaction parameters for selected 1,4-terphenyl molecules, measured by fluorescence intensity experiments under higher ionic strength conditions.

cpd[a]	*K_d (IIB_h)* (μM)	*K_d [b] (IIB_h+tRNA)* (μM)	*IIB-tRNA spec.*[c]	*K_d [b] (IIB_h+LTR_d)* (μM)	*IIB-DNA spec.*[c]	*K_d (TAR_h)* (μM)	$\frac{K_d(TAR_h)}{K_d(IIB_h)}$ [c]
**1a**	4.4 [18] (3.2-5.0 [13-20], 0.9935)	n/d	n/d	15 (12-20, 0.9523)	0.29	13 (12-15, 0.9944)	3.0
**1d**	7.6 [30] (6.1-10 [24-41], 0.9964)	42 [b] (18-120, 0.8704)	0.18	3.7 [54] (1.8-8.4 [35-74], 0.9942)	2.1	32 (28-38, 0.9873)	4.3
**3c**	>50	>50	n/d	>50	n/d	>50	n/d
**1b**	>50	>50	n/d	>50	n/d	>50	n/d

[a]For each compound (cpd), the table report best-fit IIB_h and TAR_h equilibrium dissociation constants (K_d) obtained in 10 mM HEPES (pH 7.5), 200 mM KCl and 2 mM $MgCl_2$. For binding curves best fit with a two-site model, first-site (K_d1) and second-site (K_d2; in brackets) dissociation constants are shown. For IIB_h, K_d's were measured in the absence (IIB_h) and presence of $tRNA^{Lys}$ (IIB_h+$_t$RNA) or DNA duplex LTR_d (IIB_h+LTR_d). 95% confidence intervals and R^2 coefficients are shown in parentheses. n/d: not determined.

[b]Because of the ligand concentration range used in the experiments, these curves were fit with a one-site model and the corresponding K_d values should be considered approximate.

[c]The IIB-$_t$RNA and IIB-DNA specificities of the interaction were quantified with the ratios $K_d(IIB_h)/K_d(IIB_h+{}_tRNA)$ and $K_d(IIB_h)/K_d(IIB_h+LTR_d)$, respectively. Interactions with specificity ratios close to 1 are specific, whereas those with ratios << 1 are unspecific. IIB-TAR specificities were quantified with the ratio $K_d(TAR_h)/K_d(IIB_h)$. When involving K_d1 and K_d2 values, the specificity ratios were calculated using the higher-affinity K_d1 values.

3. Synthesis and characterization of 1,4-terphenyls

With respect to NMR data, chemical shifts were referenced to the residual proton resonances of the solvents and are given in ppm (δ), and coupling constants (*J*) are given in Hertz (Hz). The letters m, s, d, t, and q stand for multiplet, singlet, doublet, triplet, and quartet, respectively, and the letters br indicate that the signal is broad.

Starting compounds **5** (4-bromo-2,5-dimethylphenol)[5], **6** (2,5-dimethyl-4-ethoxybromobenzene)[6], and **7** (4-bromo-2,5-dimethylanisole)[7] have been described previously in the literature.

Benzylic bromination

To a solution of the corresponding substrate (1 equiv) in carbon tetrachloride (0.1 M) under an atmosphere of N_2, *N*-bromosuccinimide (1.1 equiv for each benzylic position) and benzoyl peroxide (10 mol%) were added in one portion. The mixture was refluxed at 85 ˚C for 3 h and then allowed to cool to room temperature. The mixture was filtered to remove the resulting succinimide and the filtrate was concentrated *in vacuo*. The crude mixture was purified via flash column chromatography using hexane as the eluent to afford the pure product. Compounds **8b**[8] and **8c**[9] have been previously described in the literature, whereas compound **8d** is commercially available.

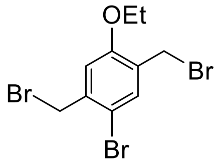

1-Bromo-2,5-bis(bromomethyl)-4-ethoxybenzene, **8a**

8a was synthesised following the general procedure for benzylic bromination starting from **7** to afford a white solid (1.26 g, 63%), with a melting point of 123-125 ˚C.

^{1}H NMR (300 MHz, CDCl$_3$) δ: 1.49 (t, *J* = 6.5 Hz, 3H), 4.13 (q, *J* = 6.8 Hz, 2H), 4.49 (s, 2H), 4.57 (s, 2H), 6.96 (s, 1H), 7.53 (s, 1H) ppm. **^{13}C NMR (75.5 MHz, CDCl$_3$) δ:** 14.7, 27.0, 33.3, 64.5, 114.3, 114.4, 128.7, 134.8, 138.2, 156.2 ppm. **HRMS (ESI):** m/z calculated for $C_{10}H_{11}Br_3O$ [M+H-Br]: 304.9171; found: 304.9170.

Nitrile synthesis

Benzyl bromide (1 equiv), sodium cyanide (1.5 equiv for each position), potassium iodide (5 mol%) and 18-crown-6 (25 mol%) were placed in a round-bottomed flask, and dissolved in a mixture of MeCN:H_2O

(8:1) (0.1 M). The resulting mixture was stirred at room temperature for 18 h, and the mixture was then diluted with water and extracted with ethyl acetate. The organic layer was washed with brine, dried over Na_2SO_4, filtered, and concentrated *in vacuo*. The crude product was purified by flash column chromatography using mixtures of hexane:EtOAc as the eluent. Compounds **9c**[10] and **9e**[11] have been previously described in the literature, and compound **9d** is commercially available.

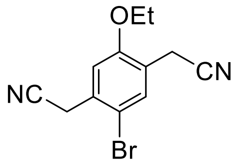

2-2'-(2-Bromo-5-ethoxy-1,4-phenylen)diacetonitrile, **9a**

9a was synthesised following the general procedure for nitrile synthesis starting from **8a** to afford a white solid (456 mg, 67%), with a melting point of 144-145 ˚C.

^{1}H NMR (300 MHz, $CDCl_3$) δ: 1.49 (t, *J* = 6.7 Hz, 3H), 3.69 (s, 2H), 3.85 (s, 2H), 4.16 (q, *J* = 7.1 Hz, 2 H), 7.06 (s, 1H), 7.60 (s, 1H) ppm. **^{13}C NMR (75.5 MHz, $CDCl_3$) δ:** 14.7, 18.4, 25.1, 64.8, 112.5, 113.6, 116.9, 117.2, 121.0, 131.0, 133.3, 156.0 ppm. **HRMS (ESI):** m/z calculated for $C_{12}H_{11}BrN_2O$ $[M+Na]^+$: 300.9947; found: 300.9946.

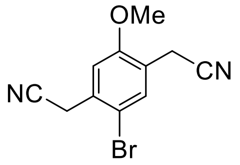

2-2'-(2-Bromo-5-methoxy-1,4-phenylen)diacetonitrile, **9b**

9a was synthesised following the general procedure for nitrile synthesis starting from **8b** to afford a white solid (624 mg, 73%), with a melting point of 143-144 ˚C.

^{1}H NMR (300 MHz, $CDCl_3$) δ: 3.68 (s, 2H), 3.85 (s, 2H), 3.92 (s, 3H), 7.07 (s, 1H), 7.60 (s, 1H) ppm. **^{13}C NMR (75.5 MHz, $CDCl_3$) δ:** 18.2, 25.0, 56.1, 111.7, 113.7, 116.7, 117.0, 120.9, 133.1, 156.5 ppm. **HRMS (ESI):** m/z calculated for $C_{11}H_9BrN_2O$ $[M+Na]^+$: 286.9790; found: 286.9787

Synthesis of 2-bromo-5-methoxyterephthalaldehyde, 10

Step 1. A mixture of **8b** (692 mg, 1.85 mmol) and calcium carbonate (926 mg, 9.25 mmol) in dioxane:water (1.4:1) (0.05 M) was refluxed at 110 ˚C for 15 h. After this time, the mixture was extracted with ethyl acetate, dried over Na_2SO_4, and concentrated *in vacuo*. The resulting crude product was used in the next step without further purification.

Step 2. The crude product from the previous step, manganese(IV) oxide (10 equiv) and dichloroethane (0.1 M) were placed in a sealed tube, and the mixture was refluxed at 100 ˚C for 12 h. The resulting mixture was filtered through a pad of Celite®, diluted with dichloromethane, and washed with water and brine. The organic phase was then dried over Na_2SO_4, filtered and concentrated *in vacuo*. The crude product was purified via flash column chromatography using mixtures of hexane:EtOAc as the eluent.

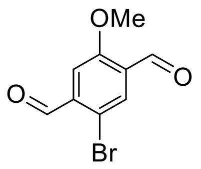

2-Bromo-5-methoxyterephthalaldehyde, **10**

10 was isolated as a white solid (396 mg, 88% from **8b**) with a melting point of 115-117 ˚C.

^{1}H NMR (300 MHz, CDCl$_3$) δ: 3.99 (s, 3H), 7.52 (s, 1H), 8.03 (s, 1H), 10.30 (s, 1H), 10.40 (s, 1H) ppm. **^{13}C NMR (75.5 MHz, CDCl$_3$) δ:** 56.5, 112.8, 117.8, 129.4, 133.7, 137.7, 160.7, 188.1, 191.4 ppm. **HRMS (ESI):** m/z calculated for $C_9H_7BrO_3$ $[M+H]^+$: 242.9651; found: 242.9647.

Synthesis of 3,3'-(2-bromo-5-methoxy-1,4-phenylene)dipropanenitrile, 11

Step 1. Cyanomethyltriphenylphosphonium bromide (1.77 g, 5.24 mmol) was added at 0 ˚C to a solution of sodium hydroxide (208 mg, 5.24 mmol) in water:dichloromethane (3:1) (0.3 M) and the resulting mixture was stirred vigorously for 15 minutes at this temperature. The starting dialdehyde (425 mg, 1.74 mmol) was then added at 0 ˚C and the reaction was allowed to warm to room temperature and left to stir for two hours. The reaction mixture was then extracted with dichloromethane and the combined organic phases were dried over Na_2SO_4, filtered and concentrated *in vacuo*.

Step 2. The crude product from the previous step was dissolved in ethanol (0.08 M) and sodium borohydride (5 equiv) was added. The mixture was then fitted with a condenser and stirred at 85 ˚C

for three hours, after which the reaction was left to cool to room temperature before quenching dropwise with water. This was then extracted with dichloromethane and the organic phases were combined and dried over Na_2SO_4, filtered and concentrated *in vacuo*. The crude mixture was then purified via flash column chromatography using mixtures of hexane:EtOAc as the eluent.

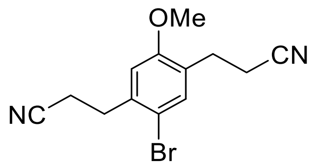

3,3'-(2-Bromo-5-methoxy-1,4-phenylene)dipropanenitrile, **11**

11 was isolated as a white solid (306 mg, 60% from **10**) with a melting point of 160-161 ˚C.

^{1}H NMR (300 MHz, $CDCl_3$) δ: 2.63 (t, *J* = 6.9 Hz, 2H), 2.69 (t, *J* = 7.1 Hz, 2H), 2.92 (t, *J* = 7.3 Hz, 2H), 3.06 (t, *J* = 6.9 Hz, 2H), 3.87 (s, 3H), 6.84 (s, 1H), 7.36 (s, 1H) ppm. **^{13}C NMR (75.5 MHz, $CDCl_3$) δ:** 17.3, 17.7, 26.4, 32.3, 55.7, 113.0, 114.0, 118.6, 119.1, 127.7, 134.1, 137.2, 156.9 ppm. **HRMS (ESI):** m/z calculated for $C_{13}H_{13}BrN_2O$ $[M+NH_4]^+$: 310.0549; found: 310.0545.

Borylation of aryl bromides

In a microwaveable vial, aryl bromide (1 equiv), bis(pinacolato)diboron (1.2 equiv), and potassium acetate (3 equiv) were dissolved in dimethoxyethane (0.2 M), and nitrogen gas was passed through the solution for 5 minutes. Subsequently, $PdCl_2$(dppf) (10 mol%) was added, the vial was sealed and placed in a microwave oven for 1 hour at 120 ˚C. After this time, the reaction was allowed to cool and filtered through a pad of Celite®, washing with dichloromethane. The crude mixture was concentrated *in vacuo* and purified via flash column chromatography using mixtures of hexane:EtOAc as the eluent. Compounds **12c**, **12d** and **12e** are commercially available, while **12f** has been previously described in the literature.[12]

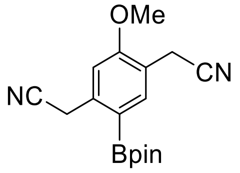

2-2’-(2-Methoxy-5-(4,4,5,5-tetramethyl-1,2,3-dioxaborolan-2-yl)phenylen)diacetonitrile, **12a**

^{1}H NMR (300 MHz, $CDCl_3$) δ: 1.34 (s, 12H), 3.65 (s, 2H), 3.93 (s, 3H), 4.12 (s, 2H), 6.98 (s, 1H), 7.80 (s, 1H) ppm. **^{13}C NMR (75.5 MHz, $CDCl_3$) δ:** 18. 4, 23.7, 25.0, 55.9, 84.3, 110.2, 110.9, 117.8, 118.0, 118.8, 138.1, 139.4, 159.6 ppm. **HRMS (ESI):** m/z calculated for $C_{17}H_{21}BN_2O_3$ $[M+NH_4]^+$: 330.1883; found: 330.1887.

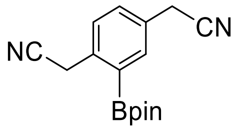

2-2'-(2-(4,4,5,5-Tetramethyl-1,2,3-dioxaborolan-2-yl)phenylen)diacetonitrile, **12b**

12b was synthesised following the general procedure for borylation of aryl bromides starting from **9c** to afford a yellow oil (312 mg, 63%).

^{1}H NMR (300 MHz, $CDCl_3$) δ: 1.36 (s, 12H), 3.75 (s, 2H), 4.09 (s, 2H), 7.46 (m, 2H), 7.81 (s, 1H) ppm. **^{13}C NMR (75.5 MHz, $CDCl_3$) δ:** 23.3, 23.4, 25.0, 84.6, 117.7, 118.7, 129.3, 129.6, 131.4, 136.5, 136.9 ppm. **HRMS (ESI):** m/z calculated for $C_{16}H_{19}BN_2O_2$ $[M+NH_4]^+$: 300.1878; found: 300.1882.

Diborylation of aryl bromides

The corresponding aryl bromide (1 equiv), potassium acetate (5 equiv) and bis(pinacolato)diboron (3 equiv) were placed in a microwave vial and anhydrous dioxane (0.2 M) was added. N_2 was bubbled through the mixture for 5 minutes, then $PdCl_2$(dppf) (10 mol%) was added. The mixture was heated at 120 ˚C for 1 h in a microwave oven, after which time the reaction mixture was allowed to cool to room temperature and filtered through a pad of Celite®, washing with dichloromethane. The filtrate was concentrated *in vacuo* and the crude mixture purified by means of flash column chromatography using mixtures of hexane:EtOAc as the eluent. Compound **13b** has been previously described in the literature.[13]

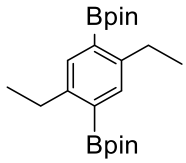

2,2'-(2,5-Diethyl-1,4-phenylene)bis(4,4,5,5-tetramethyl-1,3,2-dioxaborolane), **13a**

13a was synthesised following the general procedure for diborylation of aryl bromides starting from 4-bromo-2,5-dimethylbromobenzene to afford a pale yellow solid (402 mg, 75%) with a melting point of 204-205 ˚C.

^{1}H NMR (300 MHz, $CDCl_3$) δ: 1.17 (t, *J* = 7.4 Hz, 6H), 1.34 (s, 24H), 2.86 (q, *J* = 7.4 Hz, 4H), 7.55 (s, 2H) ppm. **^{13}C NMR (75.5 MHz, $CDCl_3$) δ:** 17.6, 25.0, 28.5, 83.5, 136.1, 147.6 ppm. **HRMS (ESI):** m/z calculated for $C_{22}H_{36}B_2O_4$ $[M+H]^+$: 387.2872; found: 387.2870.

Synthesis of biphenyls

In a microwave vial, the corresponding aryl bromide (1 equiv) and boronic ester (1.1 equiv) were dissolved in acetonitrile and N_2 was bubbled through the resulting solution for 5 minutes. A solution of K_3PO_4 (3 equiv) in water (1 mL) was then added and N_2 passed through the solution for a further minute. Finally, $PdCl_2$(dppf) (10 mol%) was added, the vial was sealed and the resulting mixture was placed in a microwave oven at 120 ˚C for 1 hour. After this time had elapsed and the reaction mixture had cooled down, water was added and the mixture was extracted with ethyl acetate. The combined organic layers were dried over Na_2SO_4, filtered and concentrated *in vacuo*. The crude mixture was purified using flash column chromatography employing mixtures of hexane:AcOEt as the eluent.

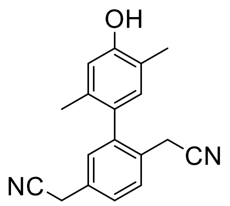

2,2'-(4'-Hydroxy-2',5'-dimethyl-[1,1'-biphenyl]-2,5-diyl)diacetonitrile, **14a**

14a was synthesised following the general procedure for biphenyl synthesis to afford a pale yellow oil (324 mg, 85%).

^{1}H NMR (300 MHz, $CDCl_3$) δ: 1.88 (s, 3H), 2.15 (s, 3H), 3.37 (s, 2H), 3.71 (s, 2H), 5.16 (br s, 1H), 6.63 (s, 1H), 6.74 (s, 1H), 7.09 (d, 1H), 7.48 (d, 1H) ppm. **^{13}C NMR (75.5 MHz, $CDCl_3$) δ:** 15.4, 19.6, 21.5, 23.4, 116.8, 117.7, 117.9, 121.8, 127.6, 128.8, 128.9, 129.4, 130.0, 130.2, 131.6, 134.5, 142.3, 154.0 ppm. **HRMS (ESI):** m/z calculated for $C_{18}H_{16}N_2O$ $[M+NH_4]^+$: 294.1601; found: 294.0607.

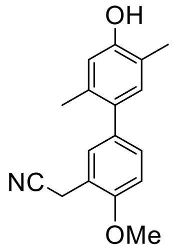

2-(4'-Hydroxy-4-methoxy-2',5'-dimethyl-[1,1'-biphenyl]-3-yl)acetonitrile, **14b**

14b was synthesised following the general procedure for biphenyl synthesis to afford a yellow oil (192 mg, 57%).

^{1}H NMR (300 MHz, $CDCl_3$) δ: 2.19 (s, 3H), 2.24 (s, 3H), 3.72 (s, 2H), 3.90 (s, 3H), 6.68 (br s, 1H), 6.91 (d, 1H), 6.95 (br s, 1H), 7.23 (dd, 1H), 7.29 (m, 1H) ppm. **^{13}C NMR (75.5 MHz, $CDCl_3$) δ:** 15.4, 18.9, 20.2, 55.8, 110.2, 116.8, 118.2, 118.3, 121.1, 130.3, 130.4, 132.6, 133.5, 134.4, 134.6, 153.1, 155.6 ppm. **HRMS (ESI):** m/z calculated for $C_{17}H_{17}NO_2$ $[M+NH_4]^+$: 285.1598; found: 285.1599.

Synthesis of triflates

Pyridine (1.5 equiv) was added to a solution of the corresponding biphenyl (1 equiv) in anhydrous dichloromethane (0.2 M) and the resulting solution was stirred for 10 minutes at 0 ˚C. Subsequently, triflic anhydride (1.5 equiv, 1 M in dichloromethane) was added and after a further 5 minutes at 0 ˚C the reaction was left to stir at room temperature for 90 minutes. After this time had elapsed, the solution was diluted with diethyl ether and aqueous HCl (3 M) was added. The organic phase was washed with water, sat. $NaHCO_3$, sat. Na_2SO_3, and brine. The organic phase was then dried over Na_2SO_4, filtered and concentrated *in vacuo*. The crude product was purified via flash column chromatography using mixtures of hexane:EtOAc as the eluent.

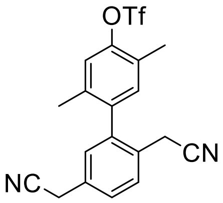

2',5'-Bis(cyanomethyl)-2,5-dimethyl-[1,1'-biphenyl]-4-yl trifluoromethanesulfonate, **15a**

15a was synthesised following the general procedure for triflate synthesis starting from **14a** to afford a colourless oil (378 mg, 95%).

^{1}H NMR (300 MHz, $CDCl_3$) δ: 2.04 (s, 3H), 2.37 (s, 3H), 3.41 (s, 2H), 3.80 (s, 2H), 7.05 (d, 1H), 7.16 (d, 1H), 7.19 (s, 1H), 7.41 (dd, 1H), 7.59 (d, 1H) ppm. **^{13}C NMR (75.5 MHz, $CDCl_3$) δ:** 16.0, 19.7, 21.2, 23.4, 117.4, 123.2, 128.4, 129.6, 129.9, 130.6, 132.8, 136.0, 138.5, 140.6, 148.2 ppm. **^{19}F NMR (282 MHz, $CDCl_3$) δ:** -73.77 (s) ppm. **HRMS (ESI):** m/z calculated for $C_{19}H_{15}N_2O_3F_3$ $[M+Na]^+$: 431.0648; found: 431.0637.

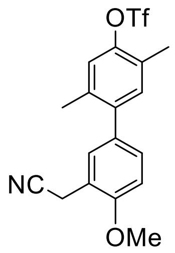

3'-(Cyanomethyl)-4'-methoxy-2,5-dimethyl-[1,1'-biphenyl]-4-yl trifluoromethanesulfonate, **15b**

15b was synthesised following the general procedure for triflate synthesis starting from **14b** to afford a colourless oil (346 mg, 89%).

^{1}H NMR (300 MHz, $CDCl_3$) δ: 2.83 (s, 3H), 2.50 (s, 3H), 3.87 (s, 2H), 4.06 (s, 3H), 7.09 (d, 1H), 7.26 (br s, 1H), 7.38 (m, 1H), 7.43 (d, 1H) ppm. **^{13}C NMR (75.5 MHz, $CDCl_3$) δ:** 16.0, 18.8, 20.3, 5.5.8, 110.4, 118.0, 118.8, 122.8, 128.0, 130.0, 130.2, 133.0, 133.5, 135.6, 141.1, 147.5, 156.2 ppm. **^{19}F NMR ($CDCl_3$, 282 MHz) δ:** -73.82 (s) ppm. **HRMS (ESI):** m/z calculated for $C_{18}H_{16}NO_4F_3$ $[M+H]^+$: 417.1090; found: 417.1099.

Synthesis of asymmetrical terphenyls

In a microwaveable vial, the corresponding triflate (1 equiv), boronic ester (1.1 equiv), and triphenylphosphine (50 mol%) were dissolved in acetonitrile (0.1 M) and N_2 gas was passed through the solution for 5 minutes. Subsequently, a solution of K_3PO_4 (3 equiv) in water (1 mL) was added and the N_2 gas was left for a further minute. Palladium acetate (10 mol%) was then added, the vial was sealed and the reaction placed in a microwave oven for 1 hour at 120 ˚C. After this time had elapsed and the reaction mixture had cooled down, water and ethyl acetate were added and the mixture extracted several times with further ethyl acetate. The combined organic layers were dried over Na_2SO_4, filtered and concentrated *in vacuo*. The crude mixture was purified via flash column chromatography using mixtures of hexane:EtOAc as the eluent. If necessary, the product was purified further via flash column chromatography employing toluene:EtOAc to remove pinacol formed by the decomposition of the boron-pinacol ester.

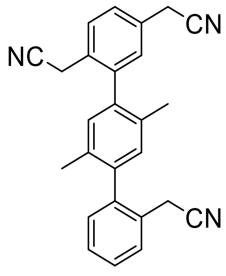

2,2',2''-(2',5'-Dimethyl-[1,1':4',1''-terphenyl]-2,2'',5-triyl)triacetonitrile, **16a**

16a was synthesised following the general procedure for asymmetric terphenyl synthesis to afford a yellow oil (121 mg, 47%).

^{1}H NMR (300 MHz, CDCl$_3$) δ: 1.95 (s, 6H), 3.41 (d, *J* = 2.4 Hz, 2H), 3.43 (d, *J* = 2.0 Hz, 2H), 3.72 (d, *J* = 2.6 Hz, 2H), 6.95 (dd, 2H), 7.07 (m, 1H), 7.16 (m, 2H), 7.33 (m, 2H), 7.49 (m, 2H) ppm. **^{13}C NMR (75.5 MHz, CDCl$_3$) δ:** 19.4, 21.6, 21.7, 21.9, 22.0, 23.4, 117.6, 117.7, 117.9, 118.0, 127.9, 128.1, 128.2, 128.3, 128.4, 128.4, 128.9, 128.8, 129.1, 129.5, 129.6, 129.7, 130.1, 130.2, 130.3, 131.0, 131.1, 131.3, 131.4, 133.3, 133.7, 133.8, 138.2, 139.5, 140.7 ppm. **HRMS (ESI):** m/z calculated for $C_{26}H_{21}N_3$ $[M+H]^+$: 376.8907; found: 376.8904.

Note: the ^{13}C spectrum for **16a** contains more signals than expected due to the presence of rotamers. This can also be seen in the splitting of several peaks in the ^{1}H spectrum.

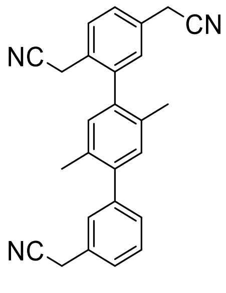

2,2',2''-(2',5'-Dimethyl-[1,1':4',1''-terphenyl]-2,3'',5-triyl)triacetonitrile, **16b**

16b was synthesised following the general procedure for asymmetric terphenyl synthesis to afford a white solid (86 mg, 39%) with a melting point of 156-157 ˚C.

^{1}H NMR (300 MHz, CDCl$_3$) δ: 1.98 (s, 3H), 2.19 (s, 3H), 3.45 (s, 2H), 3.75 (s, 2H), 3.77 (s, 2H), 6.94 (s, 1H), 7.09 (s, 1H), 7.19 (m, 1H), 7.28 (m, 3H), 7.37 (m, 2H), 7.56 (d, 1H) ppm. **^{13}C NMR (75.5 MHz, CDCl$_3$) δ:** 19.3, 19.9, 21.5, 23.3, 23.7, 117.5, 117.7, 117.8, 126.6, 127.7, 128.4, 128.7, 128.9, 129.0, 129.4,

129.6, 129.9, 130.1, 131.0, 131.7, 133.0, 133.1, 137.6, 141.1, 142.0, 142.4 ppm. **HRMS (ESI):** m/z calculated for $C_{26}H_{21}N_3$ $[M+H]^+$: 376.8907; found: 376.8904.

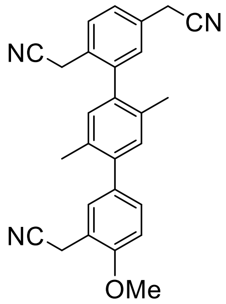

2,2',2''-(4''-Methoxy-2',5'-dimethyl-[1,1':4',1''-terphenyl]-2,3'',5-triyl)triacetonitrile, **16c**

16c was synthesised following the general procedure for asymmetric terphenyl synthesis to afford a white solid (97 mg, 57%) with a melting point of 162-163 ˚C.

^{1}H NMR (300 MHz, $CDCl_3$) δ: 2.04 (s, 3H), 2.26 (s, 3H), 3.51 (s, 2H), 3.76 (s, 2H), 3.81 (s, 2H), 3.93 (s, 3H), 6.97 (m, 2H), 7.14 (m, 1H), 7.23 (m, 1H), 7.32 (dd, 1H), 7.40 (dd, 2H), 7.61 (dd, 1H) ppm. **^{13}C NMR (75.5 MHz, $CDCl_3$) δ:** 18.9, 19.4, 20.1, 21.6, 23.4, 55.8, 110.4, 117.6, 117.8, 118.1, 118.6, 127.8, 128.4, 128.6, 129.2, 129.5, 129.8, 130.2, 131.1, 132.0, 133.0, 133.4, 134.2, 137.4, 141.1, 142.2, 156.0 ppm. **HRMS (ESI):** m/z calculated for $C_{27}H_{23}N_3O$ $[M+Na]^+$: 428.1733; found: 428.1724.

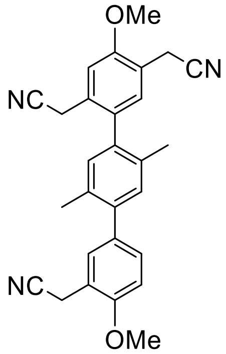

2,2',2''-(4,4''-Dimethoxy-2',5'-dimethyl-[1,1':4',1''-terphenyl]-2,3'',5-triyl)triacetonitrile, **16d**

16d was synthesised following the general procedure for asymmetric terphenyl synthesis to afford a white solid (82 mg, 59%) with a melting point of 164-166 ˚C.

^{1}H NMR (300 MHz, CD_3OD) δ: 2.04 (s, 3H), 2.25 (s, 3H), 3.51 (d, *J* = 4.0 Hz, 2H), 3.73 (s, 2H), 3.76 (s, 2H), 3.93 (s, 3H), 3.97 (s, 3H), 6.97 (m, 1H), 7.09 (s, 1H), 7.12 (m, 1H), 7.17 (m, 1H), 7.24 (m, 1H), 7.32 (dd, 1H), 7.38 (d, 1H) ppm. **^{13}C NMR (75.5 MHz, CD_3OD) δ:** 18.6, 18.9, 19.5, 20.1, 22.1, 55.8, 56.0, 110.4, 110.5, 117.8, 118.1, 118.6, 118.9, 128.3, 129.2, 129.7, 130.2, 131.0, 131.7, 132.0, 133.3, 133.5,

133.7, 134.3, 137.3, 140.9, 156.0, 156.5 ppm. **HRMS (ESI):** m/z calculated for $C_{27}H_{23}N_3O$ [M+Na+H]$^+$: 458.1839; found: 458.1842.

Synthesis of symmetrical terphenyls

In a microwaveable vial, the corresponding aryl bromide (2.1 equiv), diboronic ester (1 equiv), and triphenylphosphine (50 mol%) were dissolved in acetonitrile (0.1 M) and N_2 gas was passed through the solution for 5 minutes. Subsequently, a solution of K_3PO_4 (3.5 equiv) in water (1 mL) was added and the N_2 gas was left for a further minute. Palladium acetate (10 mol%) was then added, the vial was sealed and the reaction placed in a microwave oven for 1 hour at 120 ˚C. After this time had elapsed and the reaction mixture had cooled to room temperature, water and ethyl acetate were added and the mixture extracted several times with further ethyl acetate. The combined organic layers were dried over Na_2SO_4, filtered and concentrated *in vacuo*. The crude mixture was purified via flash column chromatography using mixtures of hexane:EtOAc as the eluent. If necessary, the product was purified further via flash column chromatography employing toluene:EtOAc to remove pinacol formed by the decomposition of the boron-pinacol ester.

The ^{13}C spectra for **compounds 17** often contain fewer signals than expected due to the complete symmetry of these structures.

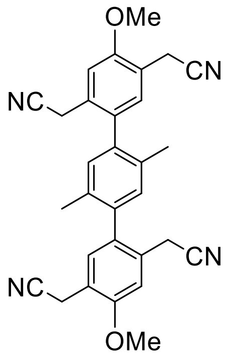

2,2',2'',2'''-(4,4''-Dimethoxy-2',5'-dimethyl-[1,1':4',1''-terphenyl]-2,2'',5,5''-tetrayl)tetraacetonitrile, **17a**

17a was synthesised following the general procedure for symmetrical terphenyl synthesis to afford a white solid (153 mg, 91%) with a melting point of 149-150 ˚C.

^{1}H NMR (300 MHz, CD_3OD) δ: 2.01 (d, 6H), 3.71 (m, 4H), 3.91 (m, 4H), 3.93 (s, 6H), 7.09 (m, 2H), 7.24 (m, 4H) ppm. **^{13}C NMR (75.5 MHz, CD_3OD) δ:** 17.6, 17.7, 19.1, 19.1, 21.1, 21.3, 55.9, 111.7, 111.8, 118.6, 118.7, 118.9, 119.0, 130.2, 130.3, 130.9, 131.0, 131.3, 131.4, 132.7, 132.8, 133.1, 133.2, 137.9, 156.2 ppm. **HRMS (ESI):** m/z calculated for $C_{30}H_{26}N_4O_2$ [M+Na+H]$^+$: 492.2394; found: 492.2384.

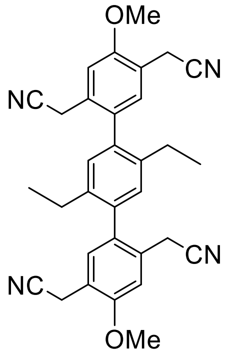

2,2',2'',2'''-(2',5'-Diethyl-4,4''-dimethoxy-[1,1':4',1''-terphenyl]-2,2'',5,5''-tetrayl)tetraacetonitrile, **17b**

17b was synthesised following the general procedure for symmetrical terphenyl synthesis to afford a white solid (122 mg, 73%) with a melting point of 153-154 ˚C.

^{1}H NMR (300 MHz, $CDCl_3$) δ: 1.05 (m, 6H), 2.33 (m, 4H), 3.51 (m, 4H), 3.74 (br s, 2H), 3.76 (br s, 2H), 3.98 (s, 6H), 7.01 (s, 1H), 7.03 (s, 1H), 7.07 (s, 1H), 7.10 (s, 1H), 7.28 (s, 1H), 7.33 (s, 1H) ppm. **^{13}C NMR (75.5 MHz, $CDCl_3$) δ:** 15.3, 18.7, 22.3, 25.9, 56.0, 110.5, 110.7, 117.8, 118.7, 118.9, 129.6, 129.9, 130.3, 130.9, 131.2, 133.3, 133.4, 138.0, 140.1, 156.5 ppm. **HRMS (ESI):** m/z calculated for $C_{32}H_{30}N_4O_2$ $[M+H]^+$: 503.6234; found: 503.6238.

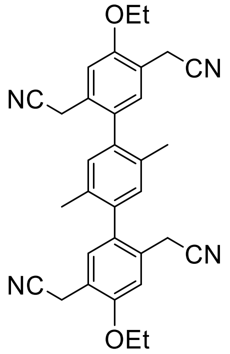

2,2',2'',2'''-(4,4''-Diethoxy-2',5'-dimethyl-[1,1':4',1''-terphenyl]-2,2'',5,5''-tetrayl)tetraacetonitrile, **17c**

17c was synthesised following the general procedure for symmetrical terphenyl synthesis to afford a pale yellow oil (78 mg, 54%).

^{1}H NMR (300 MHz, $CDCl_3$) δ: 1.51 (t, *J* =6.9 Hz, 6 H), 3.50 (m, 4H), 3.75 (m, 4H), 4.19 (q, *J* = 6.8 Hz, 4H), 6.99 (s, 1H), 7.01 (s, 1H), 7.06 (s, 1H), 7.08 (s, 1H), 7.23 (s, 1H), 7.28 (s, 1H) ppm. **^{13}C NMR (75.5 MHz, $CDCl_3$) δ:** 14.9, 18.7, 19.5, 22.0, 22.1, 64.5, 111.2, 111.4, 117.8, 117.9, 118.8, 119.1, 129.4, 129.6, 130.6,

131.0, 131.7, 133.1, 134.0, 134.1, 138.4, 155.9 ppm. **HRMS (ESI):** m/z calculated for $C_{32}H_{30}N_4O_2$ $[M+NH_4]^+$: 520.2707; found: 520.2709.

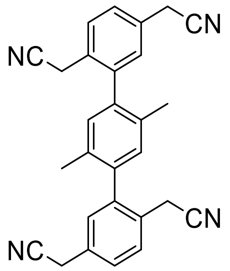

2,2',2'',2'''-(2',5'-Dimethyl-[1,1':4',1''-terphenyl]-2,2'',5,5''-tetrayl)tetraacetonitrile, **17d**

17d was synthesised following the general procedure for symmetrical terphenyl synthesis to afford a pale yellow oil (139 mg, 85%).

^{1}H NMR (300 MHz, CDCl$_3$) δ: 2.04 (s, 3H), 2.05 (s, 3H), 3.51 (d, *J* = 2.9 Hz, 4H), 3.83 (d, *J* = 2.8 Hz, 4 H), 7.04 (d, 2H), 7.24 (d, 1H), 7.28 (d, 1H), 7.40 (m, 2H), 7.60 (t, 2H) ppm. **^{13}C NMR (75.5 MHz, CDCl$_3$) δ:** 19.4, 21.7, 21.8, 23.4, 117.5, 117.7, 128.0, 128.5, 129.6, 129.6, 129.7, 129.8, 130.3, 130.4, 131.2, 131.3, 133.6, 133.7, 138.6, 141.7, 141.8 ppm. **HRMS (ESI):** m/z calculated for $C_{28}H_{22}N_4$ $[M+Na]^+$: 432.1183; found: 432.2162.

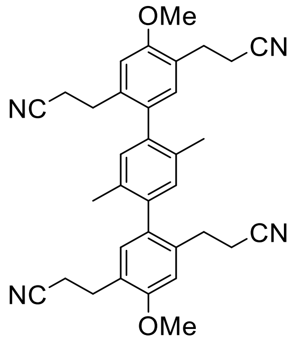

3,3',3'',3'''-(4,4''-Dimethoxy-2',5'-dimethyl-[1,1':4',1''-terphenyl]-2,2'',5,5''-tetrayl)tetrapropanenitrile, **17e**

17e was synthesised following the general procedure for symmetrical terphenyl synthesis to afford a white solid (87 mg, 63%) with a melting point of 143-144 ˚C.

^{1}H NMR (300 MHz, CDCl$_3$) δ: 2.04 (s, 3H), 2.06 (s, 3H), 2.43 (m, 4H), 2.69-3.00 (m, 12H), 3.93 (s, 6H), 6.88 (m, 2H), 7.04 (m, 4H) ppm. **^{13}C NMR (75.5 MHz, CDCl$_3$) δ:** 17.6, 18.4, 18.7, 19.7, 26.8, 29.4, 55.6,

111.3, 119.5, 119.7, 125.4, 125.5, 131.3, 131.8, 132.2, 133.3, 133.6, 133.7, 136.3, 139.1, 156.9 ppm. **HRMS (ESI):** m/z calculated for $C_{36}H_{38}N_4O_2$ $[M+NH_4]^+$: 576.3333; found: 576.3325

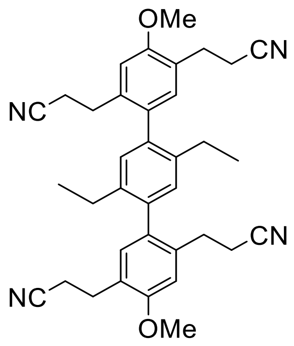

3,3',3'',3'''-(4,4''-Dimethoxy-2',5'-dimethyl-[1,1':4',1''-terphenyl]-2,2'',5,5''-tetrayl)tetrapropanenitrile, **17f**

17f was synthesised following the general procedure for symmetrical terphenyl synthesis to afford a white solid (91 mg, 66%) with a melting point of 187-189 ˚C.

^{1}H NMR (300 MHz, CDCl$_3$) δ: 1.06 (m, 8H), 2.4 (m, 9H), 2.69-2.81 (m, 9H), 3.94 (s, 6H), 6.89-7.10 (m, 6H) ppm. **^{13}C NMR (75.5 MHz, CDCl$_3$) δ:** unfortunately, we were unable to achieve an acceptable ^{13}C spectrum of compound 17f. All spectra of the subsequently reduced derivative **2b** have been identified correctly. **HRMS (ESI):** m/z calculated for $C_{36}H_{38}N_4O_2$: 576.3333 $[M+NH_4]^+$; found: 576.3325.

Reduction of nitriles

The corresponding terphenyl (1 equiv) was placed in a sealed tube in an inert atmosphere of N_2, BH_3 (1 M in THF, 25 equiv) was added, and the reaction was heated for 5 days at 85 ˚C. After this time, the reaction was allowed to cool down, and aqueous HCl (3 M, 25 equiv) was added. The mixture was then heated for 2 hours at 70 ˚C left open to air without sealing the tube. Subsequently the reaction was left to cool, transferred to a round bottom flask and concentrated. The crude reaction concentrate was dissolved in methanol and re-concentrated three times, and then purified by means of flash column chromatography using mixtures of DCM:MeOH/NH_3 (7 N) as eluents. The purified product (generally a pale yellow oil) was then immediately dissolved in methanol and HCl (2 M in ethyl ether, 10 equiv) was added and the resulting solution was left to stir for 30 minutes at room temperature. Concentration *in vacuo* then afforded the corresponding hydrochloride salt.

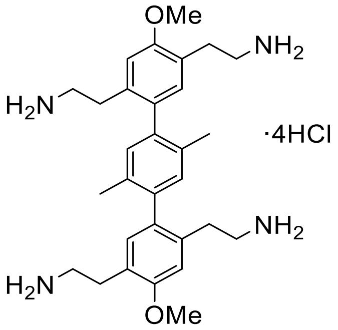

2,2',2'',2'''-(4,4''-Dimethoxy-2',5'-dimethyl-[1,1':4',1''-terphenyl]-2,2'',5,5''-tetrayl)tetrakis(ethan-1-amine) tetrahydrochloride, **1a**

1a was synthesised following the general procedure for nitrile reduction starting from **17a** to afford a white solid (24 mg, 33%) with a melting point of >300 ˚C.

^{1}H NMR (300 MHz, D_2O) δ: 1.99 (s, 6H), 2.71 (m, 2H), 2.96 (m, 10H), 3.20 (m, 4H), 3.89 (s, 6H), 7.05-7.13 (m, 6H) ppm. **^{13}C NMR (75.5MHz, CD_3OD) δ:** 18.4, 30.9, 31.0, 39.5, 39.9, 54.8, 111.4, 111.6, 118.0, 123.5, 123.6, 131.3, 131.4, 132.0, 132.1, 133.3, 133.4, 133.7, 133.9, 134.8, 135.1, 139.2, 157.2 ppm. **HRMS (ESI):** m/z calculated for $C_{30}H_{42}N_4O_2$ [M+H]$^+$: 491.3381; found: 491.3387.

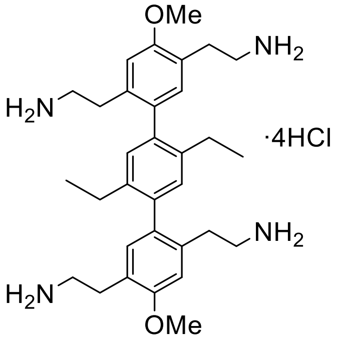

2,2',2'',2'''-(2',5'-Diethyl-4,4''-dimethoxy-[1,1':4',1''-terphenyl]-2,2'',5,5''-tetrayl)tetrakis(ethan-1-amine) tetrahydrochloride, **1b**

1b was synthesised following the general procedure for nitrile reduction starting from **17b** to afford a white solid (19 mg, 32%) with a melting point of >300 ˚C.

^{1}H NMR (300 MHz, CD_3OD) δ: 1.07 (m, 6H), 2.36-2.47 (m, 4H), 2.80-3.05 (m, 12H), 3.17 (m, 4H), 3.99 (s, 6H), 7.03-7.12 (m, 6H) ppm. **^{13}C NMR (75.5 MHz, CD_3OD) δ:** 14.4, 25.4, 28.2, 31.1, 31.2, 39.5, 39.9, 54.8, 111.3, 111.5, 123.3, 123.5, 130.0, 132.3, 132.4, 133.6, 133.8, 134.9, 135.2, 138.9, 139.4, 139.5, 157.2 ppm. **HRMS (ESI):** m/z calculated for $C_{32}H_{30}N_4O_2$ [M+H]$^+$: 503.5437; found: 503.5432.

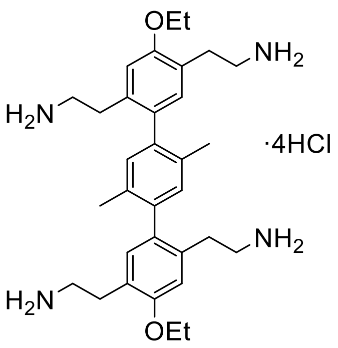

2,2',2'',2'''-(4,4''-Diethoxy-2',5'-dimethyl-[1,1':4',1''-terphenyl]-2,2'',5,5''-tetrayl)tetrakis(ethan-1-amine) tetrahydrochloride, **1c**

1c was synthesised following the general procedure for nitrile reduction starting from **17c** to afford a white solid (36 mg, 62%) with a melting point of >300 ˚C.

^{1}H NMR (300 MHz, CD$_3$OD) δ: 1.43 (t, *J* = 6.3 Hz, 6H), 2.00 (d, *J* = 2.1 Hz, 6H), 2.93 (m, 12 H), 3.14 (t, *J* = 7.9 Hz, 4H), 4.15 (q, *J* = 6.38 Hz, 4H), 6.93 (br s, 1H), 6.99 (m, 4H), 7.03 (br s, 1H) ppm. **^{13}C NMR (75.5 MHz, CD$_3$OD) δ:** 15.3, 19.8, 29.6, 32.4, 40.8, 41.3, 65.1, 113.7, 113.8, 124.8, 125.0, 132.8, 133.5, 134.6, 134.7, 135.0, 135.1, 136.1, 136.4, 140.6, 157.9 ppm. **HRMS (ESI):** m/z calculated for $C_{32}H_{46}N_4O_2$ $[M+Na]^+$: 541.3513; found: 541.3505.

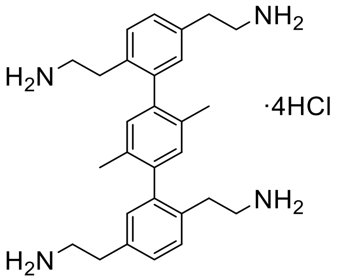

2,2',2'',2'''-(2',5'-Dimethyl-[1,1':4',1''-terphenyl]-2,2'',5,5''-tetrayl)tetrakis(ethan-1-amine) tetrahydrochloride, **1d**

1d was synthesised following the general procedure for nitrile reduction starting from **17d** to afford a white solid (17 mg, 28%) with a melting point of >300 ˚C.

^{1}H NMR (300 MHz, CD$_3$OD) δ: 2.02 (s, 6H), 2.66 (m, 8H), 2.78 (m, 4H), 2.91 (m, 4H), 7.00 (m, 4H), 7.18 (d, 1H), 7.21 (d, 1H), 7.29 (m, 2H) ppm. **^{13}C NMR (75.5 MHz, CD$_3$OD) δ:** 19.7, 30.2, 36.9, 39.4, 43.2, 43.5, 44.1, 62.8, 129.0, 130.8, 131.4, 132.2, 134.0, 136.4, 138.8, 138.9, 141.6, 142.9 ppm. **HRMS (ESI):** m/z calculated for $C_{28}H_{38}N_4$ $[M+H]^+$: 431.2169; found: 431.3158.

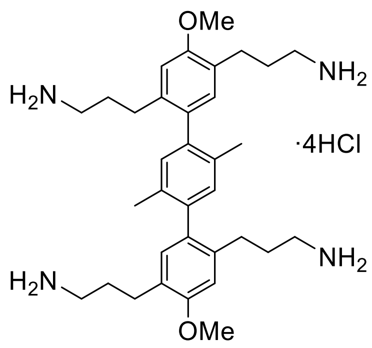

3,3',3'',3'''-(4,4''-Dimethoxy-2',5'-dimethyl-[1,1':4',1''-terphenyl]-2,2'',5,5''-tetrayl)tetrakis(propan-1-amine) tetrahydrochloride, **2a**

2a was synthesised following the general procedure for nitrile reduction starting from **17e** to afford a white solid (14 mg, 20%) with a melting point of >300 ˚C.

^{1}H NMR (300 MHz, CD_3OD) δ: 1.92 (m, 8H), 2.03 (s, 6H), 2.47 (m, 2H), 2.61 (m, 2H), 2.74 (m, 8H), 2.93 (m, 4H), 3.92 (s, 6H), 6.96 (m, 6H) ppm. **^{13}C NMR (75.5 MHz, CD_3OD) δ:** 14.4, 19.9, 23.7, 27.8, 29.1, 30.0, 31.1, 40.1, 56.1, 112.2, 127.9, 132.5, 132.6, 132.7, 134.5, 134.6, 134.7, 139.0, 139.1, 141.0, 158.2 ppm. **HRMS (ESI):** m/z calculated for $C_{34}H_{50}N_4O_2$ $[M+H]^+$: 546.2864; found: 546.2849.

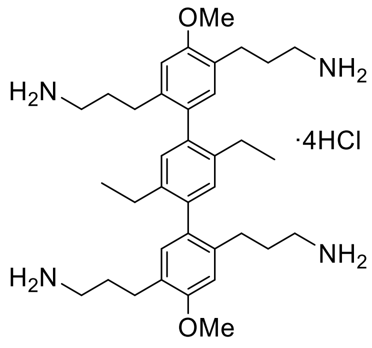

3,3',3'',3'''-(2',5'-Diethyl-4,4''-dimethoxy-[1,1':4',1''-terphenyl]-2,2'',5,5''-tetrayl)tetrakis(propan-1-amine) tetrahydrochloride, **2b**

2b was synthesised following the general procedure for nitrile reduction starting from **17f** to afford a white solid (16 mg, 24%) with a melting point of >300 ˚C.

^{1}H NMR (300 MHz, CD_3OD) δ: 1.03 (br s, 6H), 1.94 (m, 8H), 2.42 (br s, 4H), 2.58 (br s, 4H), 2.78 (m, 8H), 2.96 (br s, 4H), 3.93 (s, 6H), 7.08-6.90 (m, 6H) ppm. **^{13}C NMR (75.5 MHz, CD_3OD) δ:** 16.1, 26.9, 27.8, 29.0, 30.0, 31.2, 40.7, 56.3, 112.1, 127.6, 131.3, 132.7, 132.8, 134.3, 134.4, 139.0, 139.1, 140.6, 140.7, 158.2 ppm. **HRMS (ESI):** m/z calculated for $C_{36}H_{54}N_4O_2$ $[M+H]^+$: 575.4320; found: 575.4313.

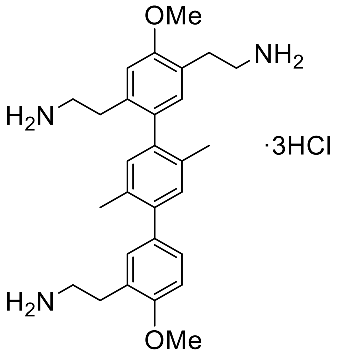

2,2',2''-(4,4''-Dimethoxy-2',5'-dimethyl-[1,1':4',1''-terphenyl]-2,3'',5-triyl)tris(ethan-1-amine) trihydrochloride, **3a**

3a was synthesised following the general procedure for nitrile reduction starting from **16d** to afford a pale yellow solid (23 mg, 52%) with a melting point of >300 ˚C.

^{1}H NMR (300 MHz, CD_3OD) δ: 2.07 (s, 3H), 2.27 (s, 3H), 2.80 (m, 1H), 2.98-3.09 (m, 7H), 3.20 (m, 4H), 3.95 (s, 3H), 3.98 (s, 3H), 7.02 (s, 1H), 7.06-7.12 (m, 3H), 7.15 (s, 1H), 7.26 (m, 2H) ppm. **^{13}C NMR (75.5 MHz, CD_3OD) δ:** 19.7, 20.3, 29.6, 30.0, 32.5, 40.8, 41.0, 41.3, 56.1, 56.2, 111.6, 112.9, 125.0, 125.8, 130.6, 132.5, 132.6, 133.0, 133.9, 134.7, 135.3, 135.6, 136.2, 140.1, 142.0, 158.2, 158.6 ppm. **HRMS (ESI):** m/z calculated for $C_{28}H_{37}N_3O_2$ $[M+NH_4]^+$: 465.8796; found: 465.8801.

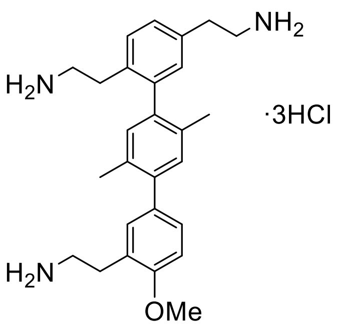

2,2',2''-(4''-Methoxy-2',5'-dimethyl-[1,1':4',1''-terphenyl]-2,3'',5-triyl)tris(ethan-1-amine) trihydrochloride, **3b**

3b was synthesised following the general procedure for nitrile reduction starting from **16c** to afford a white solid (19 mg, 39%) with a melting point of >300 ˚C.

^{1}H NMR (300 MHz, CD_3OD) δ: 1.99 (s, 3H), 2.20 (s, 3H), 2.95 (m, 8H), 3.13 (m, 4H), 7.00 (d, *J* = 5.7 Hz, 1H), 7.04 (m, 2H), 7.09 (br s, 1H), 7.18 (m, 2H), 7.27 (dd, 1H), 7.35 (d, 1H) ppm. **^{13}C NMR (75.5 MHz, CD_3OD) δ:** 19.6, 20.3, 30.0, 31.9, 34.1, 34.1, 40.9, 41.2, 41.9, 56.1, 111.7, 125.8, 129.4, 130.6, 131.1, 131.7, 132.5, 132.7, 133.1, 133.9, 134.2, 134.7, 135.5, 137.1, 137.3, 140.2, 142.2, 143.5, 158.2 ppm. **HRMS (ESI):** m/z calculated for $C_{27}H_{35}N_3O$: 419.3169 $[M+H]^+$; found: 419.3149.

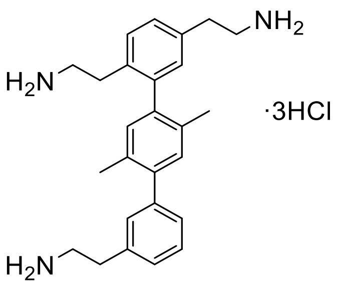

2,2',2''-(2',5'-Dimethyl-[1,1':4',1''-terphenyl]-2,3'',5-triyl)tris(ethan-1-amine) trihydrochloride, **3c**

3c was synthesised following the general procedure for nitrile reduction starting from **16b** to afford a white solid (27 mg, 40%) with a melting point of >300 ˚C.

^{1}H NMR (300 MHz, CD_3OD) δ: 2.08 (s, 3H), 2.28 (s, 3H), 2.76-3.08 (m, 8H), 3.23 (m, 4H), 7.10 (s, 1H), 7.13 (s, 1H), 7.19 (s, 1H), 7.32 (m, 4H), 7.45 (m, 2H) ppm. **^{13}C NMR (75.5 MHz, CD_3OD) δ:** 14.0, 18.2, 18.8, 32.7, 33.2, 39.8, 40.6, 40.7, 127.0, 127.7, 128.1, 128.5, 129.3, 129.7, 130.3, 131.1, 131.2, 132.4, 132.9, 133.2, 135.7, 136.7, 139.2, 141.1, 142.0, 142.2 ppm. **HRMS (ESI):** m/z calculated for $C_{26}H_{33}N_3$ $[M+H]^+$: 388.2747; found: 388.2729.

Note: the spectra of 3c contain small amounts of diethyl ether from precipitation.

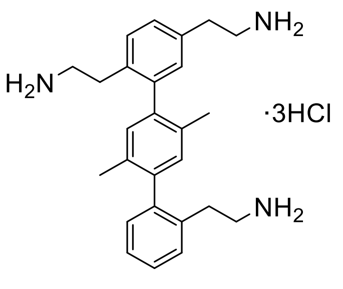

2,2',2''-(2',5'-Dimethyl-[1,1':4',1''-terphenyl]-2,2'',5-triyl)tris(ethan-1-amine) trihydrochloride, **4**

4 was synthesised following the general procedure for nitrile reduction starting from **16a** to afford a white solid (25 mg, 58%) with a melting point of >300 ˚C.

^{1}H NMR (300 MHz, CD_3OD) δ: 2.02 (s, 6H), 2.67 (m, 8H), 2.78 (m, 2H), 2.91 (m, 2H), 6.99 (t, 1H), 7.02 (d, 2H), 7.11 (m, 1H), 7.19 (dd, 1H), 7.25 (dd, 1H), 7.32 (m, 3H) ppm. **^{13}C NMR (75.5 MHz, CD_3OD) δ:** 19.8, 37.0, 37.4, 39.5, 43.3, 43.5, 44.2, 127.3, 128.6, 129.0, 130.6, 130.8, 130.9, 131.4, 132.2, 134.0, 136.3, 136.4, 138.4, 138.5, 138.8, 138.9, 141.6, 142.7, 142.9 ppm. **HRMS (ESI):** m/z calculated for $C_{26}H_{33}N_3$ $[M+H]^+$: 388.2747; found: 388.2749.

4. References

1 Battiste, J. L. *et al.* a Helix-RNA Major Groove Recognition in an HIV-1 Rev Peptide-RRE RNA Complex. *Science* **273**, 1547-1551 (1996).

2 Kjems, J., Calnan, B. J., Frankel, A. D. & Sharp, P. A. Specific binding of a basic peptide from HIV-1 Rev. *EMBO J.* **11**, 1119-1129 (1992).

3 Tan, R., Chen, L., Buettner, J. A., Hudson, D. & Frankel, A. D. RNA recognition by an isolated alpha helix. *Cell* **73**, 1031-1040 (1993).

4 Gonzalez-Bulnes, L. *et al.* Structure-Based Design of an RNA-Binding p-Terphenylene Scaffold that Inhibits HIV-1 Rev Protein Function. *Angew. Chem. Int. Ed.* **52**, 13405-13409 (2013).

5 Johansson, D. M. *et al.* Synthesis of Soluble Phenyl-Substituted Poly(*p*-phenylenevinylenes) with a Low Content of Structural Defects. *Macromolecules* **35**, 4997−5003 (2002).

6 Holmwood, G. *et al.* Heterocycle Amide Derivatives. Int. Pat. Appl. WO 2003004464, January 16, 2003.

7 Lee, J.-I.; Shim, H.-K.; Lee, G.-J.; Kim, D., Synthesis of Poly(2-methoxy-5-methyl-1,4-phenylenevinylene) and Its Poly(1,4-phenylenevinylene) Copolymers: Electrical and Third-Order Nonlinear Optical Properties. *Macromolecules* **28**, 4675-4680 (1995).

8 Jin, J.-I.; Kim, J.-C.; Shim, H.-K., Synthesis and electrical properties of poly(2-bromo-5-methoxy-1,4-phenylenevinylene) and copolymers. *Macromolecules* **25**, 5519-5523 (1992).

9 Bhasker, R.; Mindy, L., A green bromination method for the synthesis of benzylic dibromides. *Tetrahedron Lett.* **55**, 4905-4908 (2014).

10 Wheland, R. C.; Martin, E. L., Synthesis of substituted 7,7,8,8-tetracyanoquinodimethanes. *J. Org. Chem.* **40**, 3101-09 (1975).

11 Langlois, M. *et al.*Design and synthesis of new naphthalenic derivatives as ligands for 2-[125I]iodomelatonin binding sites. *J. Med. Chem.* **38**, 2050-2060 (1995).

12 Schulz, M. J.; Coats, S. J.; Hlasta, D. J., Microwave-assisted preparation of aryltetrazoleboronate esters. *Org. Lett.* **6**, 3265-3268 (2004).

13 Burke, M. J.; Nichol, G. S.; Lusby, P. J., Orthogonal Selection and Fixing of Coordination Self-Assembly Pathways for Robust Metallo-organic Ensemble Construction. *J. Am. Chem. Soc.* **138**, 9308-9315 (2016).
